# IL-7–dependent and –independent lineages of IL-7R–dependent human T cells

**DOI:** 10.1172/JCI180251

**Published:** 2024-10-01

**Authors:** Carlos A. Arango-Franco, Masato Ogishi, Susanne Unger, Ottavia M. Delmonte, Julio César Orrego, Ahmad Yatim, Margarita M. Velasquez-Lopera, Andrés F. Zea-Vera, Jonathan Bohlen, Marwa Chbihi, Antoine Fayand, Juan Pablo Sánchez, Julian Rojas, Yoann Seeleuthner, Tom Le Voyer, Quentin Philippot, Kathryn J. Payne, Adrian Gervais, Lucia V. Erazo-Borrás, Luis A. Correa-Londoño, Axel Cederholm, Alejandro Gallón-Duque, Pedro Goncalves, Jean-Marc Doisne, Liran Horev, Bénédicte Charmeteau-de Muylder, Jesús Á. Álvarez, Diana M. Arboleda, Lizet Pérez-Zapata, Estefanía Vásquez-Echeverri, Marcela Moncada-Vélez, Juan A. López, Yolanda Caicedo, Boaz Palterer, Pablo J. Patiño, Carlos J. Montoya, Matthieu Chaldebas, Peng Zhang, Tina Nguyen, Cindy S. Ma, Mohamed Jeljeli, Juan F. Alzate, Felipe Cabarcas, Taushif Khan, Darawan Rinchai, Jean-Luc Prétet, Bertrand Boisson, Generalized Verrucosis Japanese Consortium, Nico Marr, Ruba Ibrahim, Vered Molho-Pessach, Stéphanie Boisson-Dupuis, Dimitra Kiritsi, João T. Barata, Nils Landegren, Bénédicte Neven, Laurent Abel, Andrea Lisco, Vivien Béziat, Emmanuelle Jouanguy, Jacinta Bustamante, James P. Di Santo, Stuart G. Tangye, Luigi D. Notarangelo, Rémi Cheynier, Ken Natsuga, Andrés A. Arias, José Luis Franco, Klaus Warnatz, Jean-Laurent Casanova, Anne Puel

**Affiliations:** 1 Laboratory of Human Genetics of Infectious Diseases, Necker Branch, INSERM U1163, Necker Hospital for Sick Children, Paris, France.; 2 Paris Cité University, Imagine Institute, Paris, France.; 3 Inborn Errors of Immunity Group, (Primary Immunodeficiencies), School of Medicine, University of Antioquia UdeA, Medellín, Colombia.; 4 St. Giles Laboratory of Human Genetics of Infectious Diseases, Rockefeller Branch, The Rockefeller University, New York, New York, USA.; 5 Department of Rheumatology and Clinical Immunology and; 6 Center for Chronic Immunodeficiency, University Medical Center Freiburg, Faculty of Medicine, University Freiburg, Freiburg, Germany.; 7 Laboratory of Clinical Immunology and Microbiology, National Institute of Allergy and Infectious Diseases, NIH, Bethesda, Maryland, USA.; 8 Sección de Dermatología, Facultad de Medicina, Universidad de Antioquia, Centro de Investigaciones Dermatológicas (CIDERM), Medellín, Antioquia, Colombia.; 9 Clinical Immunology Clinic, Hospital Universitario del Valle, Cali, Colombia.; 10 Microbiology Department, Universidad del Valle, Cali, Colombia.; 11 Pediatric Immunology, Hematology and Rheumatology Department, Hôpital Necker-Enfants Malades, Assistance Publique-Hôpitaux de Paris, Paris, France.; 12 Microbiology School, University of Antioquia UdeA, Medellín, Colombia.; 13 Science for Life Laboratory, Department of Medical Biochemistry and Microbiology, Uppsala University, Uppsala, Sweden.; 14 Innate Immunity Unit, Institut Pasteur, Paris, France.; 15 INSERM U1223, Paris, France.; 16 Faculty of Medicine, Hebrew University of Jerusalem, Pediatric Dermatology Service, Department of Dermatology, Hadassah Medical Center, Jerusalem, Israel.; 17 Shamir (Assaf Harofeh) Medical Center, Be’er Ya’akov, Israel.; 18 Université Paris Cité, CNRS, INSERM, Institut Cochin, Paris, France.; 19 Infectología Pediátrica, Clinica Farallones, Cali, Colombia.; 20 School of Medicine, University of Antioquia UdeA, Medellin, Colombia.; 21 Garvan Institute of Medical Research, Darlinghurst, New South Wales, Australia.; 22 School of Clinical Medicine, Faculty of Medicine and Health, UNSW Sydney, Sydney, Australia.; 23 Cochin University Hospital, Biological Immunology Unit, AP-HP, Paris, France.; 24 Centro Nacional de Secuenciación Genómica CNSG, Universidad de Antioquia UdeA, Medellín, Colombia.; 25 Research Branch, Sidra Medicine, Doha, Qatar.; 26 College of Health and Life Sciences, Hamad Bin Khalifa University, Doha, Qatar.; 27 Université de Franche-Comté, CNRS, Chrono-environnement & CHU Besançon, Centre National de Référence Papillomavirus, F-25000 Besançon, France.; 28 See Supplemental Acknowledgments for the Generalized Verrucosis Japanese Consortium details.; 29 Department of Dermatology, University Medical Center of Freiburg, Freiburg, Germany.; 30 Instituto de Medicina Molecular João Lobo Antunes, Faculdade de Medicina, Universidade de Lisboa, Lisboa, Portugal.; 31 Centre for Molecular Medicine, Department of Medicine (Solna), Karolinska Institute, Stockholm, Sweden.; 32 Laboratory of Immunoregulation, National Institute of Allergy and Infectious Diseases, NIH, Bethesda, Maryland, USA.; 33 Center for the Study of Primary Immunodeficiencies, Necker Hospital for Sick Children, Paris, France.; 34 Department of Dermatology, Faculty of Medicine and Graduate of Medicine, Hokkaido University, Sapporo, Japan.; 35 Department of Pediatrics, Necker Hospital for Sick Children, AP-HP, Paris, France.; 36 Howard Hughes Medical Institute, New York, New York, USA.

**Keywords:** Genetics, Immunology, Cytokines, Genetic diseases, T cell development

## Abstract

Infants with biallelic *IL7R* loss-of-function variants have severe combined immune deficiency (SCID) characterized by the absence of autologous T lymphocytes, but normal counts of circulating B and NK cells (T^–^B^+^NK^+^ SCID). We report 6 adults (aged 22 to 59 years) from 4 kindreds and 3 ancestries (Colombian, Israeli Arab, Japanese) carrying homozygous *IL7* loss-of-function variants resulting in combined immunodeficiency (CID). Deep immunophenotyping revealed relatively normal counts and/or proportions of myeloid, B, NK, and innate lymphoid cells. By contrast, the patients had profound T cell lymphopenia, with low proportions of innate-like adaptive mucosal-associated invariant T and invariant NK T cells. They also had low blood counts of T cell receptor (TCR) excision circles, recent thymic emigrant T cells and naive CD4^+^ T cells, and low overall TCR repertoire diversity, collectively indicating impaired thymic output. The proportions of effector memory CD4^+^ and CD8^+^ T cells were high, indicating IL-7–independent homeostatic T cell proliferation in the periphery. Intriguingly, the proportions of other T cell subsets, including TCRγδ^+^ T cells and some TCRαβ^+^ T cell subsets (including Th1, Tfh, and Treg) were little affected. Peripheral CD4^+^ T cells displayed poor proliferation, but normal cytokine production upon stimulation with mitogens in vitro. Thus, inherited IL-7 deficiency impairs T cell development less severely and in a more subset-specific manner than IL-7R deficiency. These findings suggest that another IL-7R–binding cytokine, possibly thymic stromal lymphopoietin, governs an IL-7–independent pathway of human T cell development.

## Abstract 2

Homozygous IL7 loss-of-function variants in adults with combined immunodeficiency, marked by low thymic output, T-cell lymphopenia, and alteration of some T-cell subset

## Graphical abstract

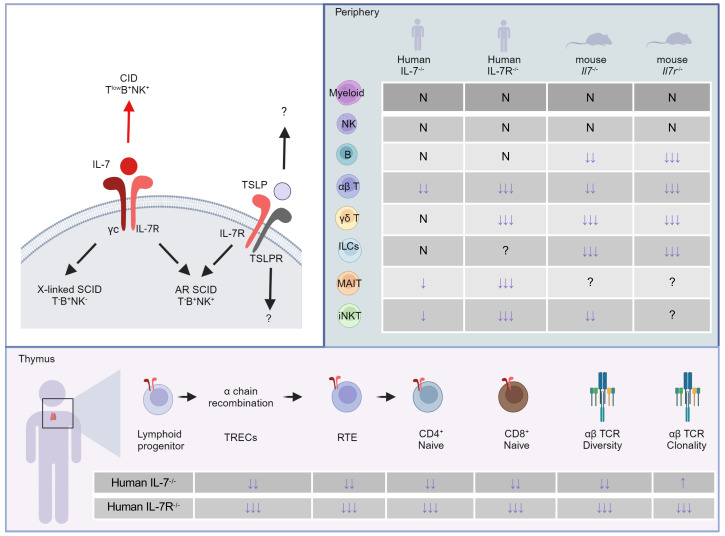

## Introduction

Genetic abnormalities of T lymphocyte–intrinsic development or function can underlie severe combined immunodeficiency (SCID), a life-threatening condition that is fatal unless treated by hematopoietic stem cell transplantation or gene therapy within the first year of life (1–3). SCID patients are typically susceptible to recurrent and life-threatening diseases caused by viruses, bacteria, fungi, and parasites early in life (1). Nineteen known inborn errors of immunity cause SCID by affecting T cell commitment at the early progenitor stages (e.g., *IL7R*, *IL2RG*, and *JAK3*), T cell survival (e.g., *AK2*, *ADA*, and *RAC2*), T cell receptor (TCR) recombination (e.g., *RAG1*, *RAG2*, *LIG4*, *PRKDC*, *DCLRE1C*, and *NHEJ1*), and pre-TCR/TCR signaling (e.g., *CD45*, *CD3D*, *CD3E*, *CD3Z*, *SLP76*, *LAT*, and *CORO1A*) (3). Approximately one-third of SCID cases are due to hemizygosity for variants of *IL2RG*, encoding the cytokine receptor γ-chain (γ_c_) common to the receptors of IL-2, IL-4, IL-7, IL-9, IL-15, and IL-21 (4–8). Human X-linked γ_c_ deficiency results in SCID lacking T and NK cells, but not B cells (T^–^B^+^NK^–^ SCID), a phenotype also observed in individuals with autosomal recessive (AR) deficiencies of JAK3, which functions downstream of γ_c_ (9, 10). B cells are present, but their activation and differentiation are compromised, reflecting impaired T cell help through IL-21–mediated signaling (4, 9–14). The first report, in 1998, of SCID patients with biallelic deleterious variants of *IL7R* lacking T, but not B or NK cells (T^–^B^+^NK^+^ SCID), strongly suggested that the absence of T cells in patients with γ_c_ or JAK3 deficiency resulted from impaired IL-7 signaling (15). AR IL-7R deficiency accounts for 5%–11% of the SCID cases diagnosed through newborn screening (1, 12, 13, 15–18).

T cells are derived from the common lymphoid progenitors (CLPs), which differentiate into pre-T cells in the bone marrow. These pre-T cells then migrate to the thymus as early thymocyte precursors (ETPs) (19). In mice, ETPs generate CD4^–^CD8^–^ double-negative (DN) cells. The divergence between αβ and γδ T cells occurs early during DN cell differentiation, with stochastic rearrangement and allelic exclusion at the *TCRB*, *TCRD*, and *TCRG* loci (19–21). Thymocytes in which the *TCRD* and *TCRG* loci are successfully rearranged express a functional γδ TCR and undergo proliferation to become functionally mature γδ T cells, whereas those with productive rearrangements at the *TCRB* locus heterodimerize with the pre-TCR-α protein, leading to proliferation and differentiation into CD4^+^CD8^+^ double-positive (DP) thymocytes. At this stage, *TCRG* expression is silenced, and *TCRA* rearrangement begins (20). DP thymocytes with functional TCRαβ receptors undergo positive selection in the cortex, negative selection in the medulla, and exit the thymus as CD4^+^ or CD8^+^ single-positive (SP) cells (22). CLPs also give rise to B cells in the bone marrow by differentiating sequentially into pro-B cells, pre-B cells, and then immature B cells, which exit the bone marrow as transitional B cells to develop into mature B cells in the periphery (23, 24). During T and B cell lymphopoiesis, *IL7R* expression, which occurs predominantly in lymphoid cells, is tightly regulated (24, 25). In addition to heterodimerizing with γ_c_ to mediate IL-7 signaling (8, 26), IL-7R also heterodimerizes with cytokine receptor–like factor 2 (CRLF2) to mediate thymic stromal lymphopoietin (TSLP) signaling (27, 28). No deleterious variants of human *TSLP* or *CRLF2* have been reported (3), but we previously identified 3 patients with AR IL-7 deficiency (29), prompting further investigation into the role of IL-7 in T cell development and human immunity through an in-depth analysis of these 3 patients, including the screening of our database of over 23,000 exomes for additional patients carrying rare predicted deleterious biallelic variants of *IL7*.

## Results

### Six patients from 4 kindred homozygous for private variants of IL7.

**Figure 1:**
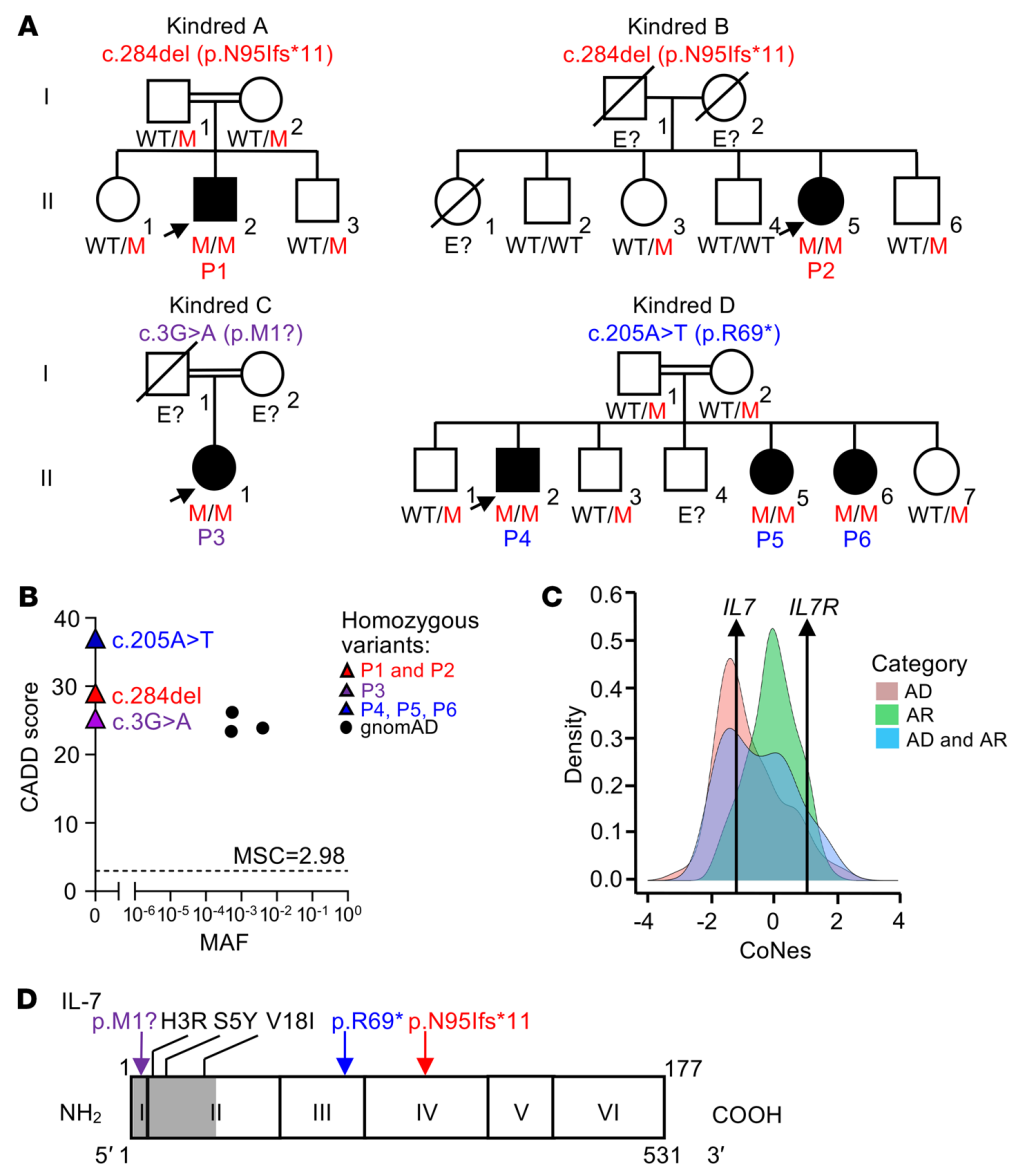
Private biallelic *IL7* variants in 6 patients from 4 unrelated kindred. (**A**) Family pedigrees with allele segregation. The patients, in black, suffer from CID and are homozygous for the indicated *IL7* alleles. The arrow indicates the proband. Each generation is designated by a Roman numeral (I, II), individuals of unknown genotype are indicated by “E?”. M, mutated; WT, wild-type. (**B**) Combined annotation depletion-dependent (CADD) score v1.6 (*y* axis) versus minor allele frequency (MAF) (*x* axis) plot for all nonsynonymous *IL7* variants present in the homozygous state in the gnomAD database v2.1 (black dots), and the 3 *IL7* variants of P1–P6 (P1 and P2, c.284del/p.N95Ifs*11, red triangle; P3, c.3G>A/p.M1?, purple triangle; and P4–P6, c.205A>T/p.R69*, blue triangle). The 99% mutation significance cutoff (MSC) is indicated (dotted line). (**C**) Consensus negative selection (CoNeS) of *IL7* and *IL7R*. AR, autosomal recessive; AD, autosomal dominant. (**D**) Schematic representation of the IL-7 protein. Exons are indicated by Roman numerals. The signal peptide is highlighted in gray (amino acids 1–25). The positions of the variants found in the patients are indicated (P1 and P2 in red; P3 in purple, and P4–P6 in blue), together with the positions of the variants found in the homozygous state in gnomAD (in black).

We searched for nonsynonymous, essential splice site, or copy number variants of *IL7* by screening our in-house whole-exome sequencing/whole-genome sequencing (WES/WGS) database of over 23,000 patients with various infectious diseases. We selected rare predicted deleterious variants present in the homozygous or compound heterozygous state with a minor allele frequency (MAF) of less than 0.01. We identified 3 unrelated patients (P1–P3) from 3 kindreds, homozygous for private, predicted loss-of-function (pLOF) *IL7* variants (Figure 1A, and Supplemental Figure 1, A and B; supplemental material available online with this article; https://doi.org/10.1172/JCI180251DS1). The patients suffered from various infectious diseases (Supplemental Table 1; clinical details in Supplemental Methods). Both P1 (Kindred A) and P2 (Kindred B) were homozygous for a single nucleotide deletion, c.284del, in exon 4 of *IL7*, leading to a frameshift variant (p.N95Ifs*11, hereafter referred to as p.N95fs) (NM_000880.4 GRCh37) (Figure 1A). WES, WGS, and a genome-wide association study (GWAS) showed that P1 and P2 had a 21.67 Mb haplotype in common, suggesting a common ancestor, estimated 12 generations or approximately 300 years ago, according to ESTIAGE software (https://lysine.univ-brest.fr/estiage/) (Supplemental Figure 1C). P3 (Kindred C) was homozygous for a variant of *IL7* at the start codon (c.3G>A/p.M1?) in exon 1 (Figure 1A). Sanger sequencing confirmed that P1–P3 were homozygous, whereas asymptomatic relatives for whom DNA was available were either heterozygous or homozygous WT, consistent with an AR trait (Figure 1A). The Colombian and Japanese ancestry of these patients was confirmed by principal component analysis (PCA) on the WES data (Supplemental Figure 1D). We also included P4–P6 (Kindred D) born to a consanguineous Arab family presenting with infectious diseases, as reported in a previous study (29) (Supplemental Table 1 and Figure 1A; clinical details in Supplemental Methods). Briefly, WES on P4 identified a biallelic nonsense variant of *IL7* (c.205A>T) in exon 3 leading to a premature stop codon (p.R69*). This variant was confirmed by Sanger sequencing, and found to be present in the homozygous state in P5 and P6, whereas the unaffected family members were heterozygous for the variant (29) (Figure 1A). No other *IL7* variants were found in the homozygous or compound heterozygous state in our in-house WES/WGS database. None of the 3 *IL7* variants was found in the homozygous or heterozygous state in public databases, including the Genome Aggregation Database (gnomAD) v2.1.1 (https://gnomad.broadinstitute.org/gene/ENSG00000104432?dataset=gnomad_r2_1), ExAC v1.0 (https://gnomad.broadinstitute.org/gene/ENSG00000104432?dataset=exac), 1000 Genomes (http://grch37.ensembl.org), BRAVO (https://bravo.sph.umich.edu/freeze8/hg38), and ATAVDB (http://atavdb.org). The 3 variants present in the 6 patients have high gene damage prediction (combined annotation–dependent depletion [CADD]) scores of 29 (c.284del), 25 (c.3G>A), and 37 (c.205A>T), well above the 99% mutation significance cutoff of 2.98 (Figure 1B). These data strongly suggest that the 6 patients have AR IL-7 deficiency.

### Population genetics of IL7, TSLP, and their receptors IL7R, IL2RG, and CRLF2.

No pLOF and only 3 missense (c.8A>G/p.H3R, c.14C>A/p.S5Y, and c.52G>A/p.V18I) *IL7* variants were found in the homozygous state in gnomAD v2.1, with a MAF of 5.2 × 10^–4^, 5.5 × 10^–4^, and 4 × 10^–3^, respectively (Figure 1B). Consistent with these findings, the *IL7* locus is known to be subject to strong purifying selection, with a consensus negative selection (CoNeS) score of –1.2 (30) (Figure 1C). Similarly, no pLOF variants of *IL7R* were found in the homozygous state, whereas 15 missense *IL7R* variants were reported in the homozygous state in the gnomAD database, with MAF values ranging from 6.4 × 10^–5^ to 6.5 × 10^–1^ (Supplemental Figure 1E). The *IL7R* locus appears to be under weaker selection pressure than the *IL7* locus, with a CoNeS score of 1.1, consistent with its reported AR inheritance (15, 17) (Figure 1C and Supplemental Figure 1F). No pLOF variant of the canonical transcript of *IL2RG,* encoding γ_c_, the other subunit of IL-7R, was found in the homozygous or hemizygous state in the general population. Only 4 missense *IL2RG* variants were found in the homozygous state in gnomAD, with MAF values ranging from 1.59 × 10^–4^ to 2.99 × 10^–3^, and 55 variants were found in the hemizygous state in gnomAD, with MAF values ranging from 5.45 × 10^–6^ to 2.99 × 10^–3^ (Supplemental Figure 1G). IL-7R also heterodimerizes with CRLF2 to form the TSLP receptor. No *TSLP* pLOF variants were found in the homozygous state in the general population, whereas only 1 missense variant (p.Cys3Ser), affecting a noncanonical *TSLP* transcript, was found in the homozygous state in gnomAD, with a MAF of 2.5 × 10^–4^ (Supplemental Figure 1H). The *TSLP* locus is subject to purifying selection, but to a lesser extent than the *IL7* locus, with a CoNeS score of –0.22 (Supplemental Figure 1F). Finally, no pLOF or hemizygous variants of *CRLF2* were identified, but 5 missense variants of *CRLF2* were found in the homozygous state, with MAF values ranging from 3.68 × 10^–4^ to 6.09 × 10^–2^, in the gnomAD database (Supplemental Figure 1I). Collectively, these findings suggest that *IL7* is under strong purifying selection and that the patients’ variants are deleterious.

### The patients’ IL7 alleles are loss-of-expression in an overexpression system.

**Figure 2:**
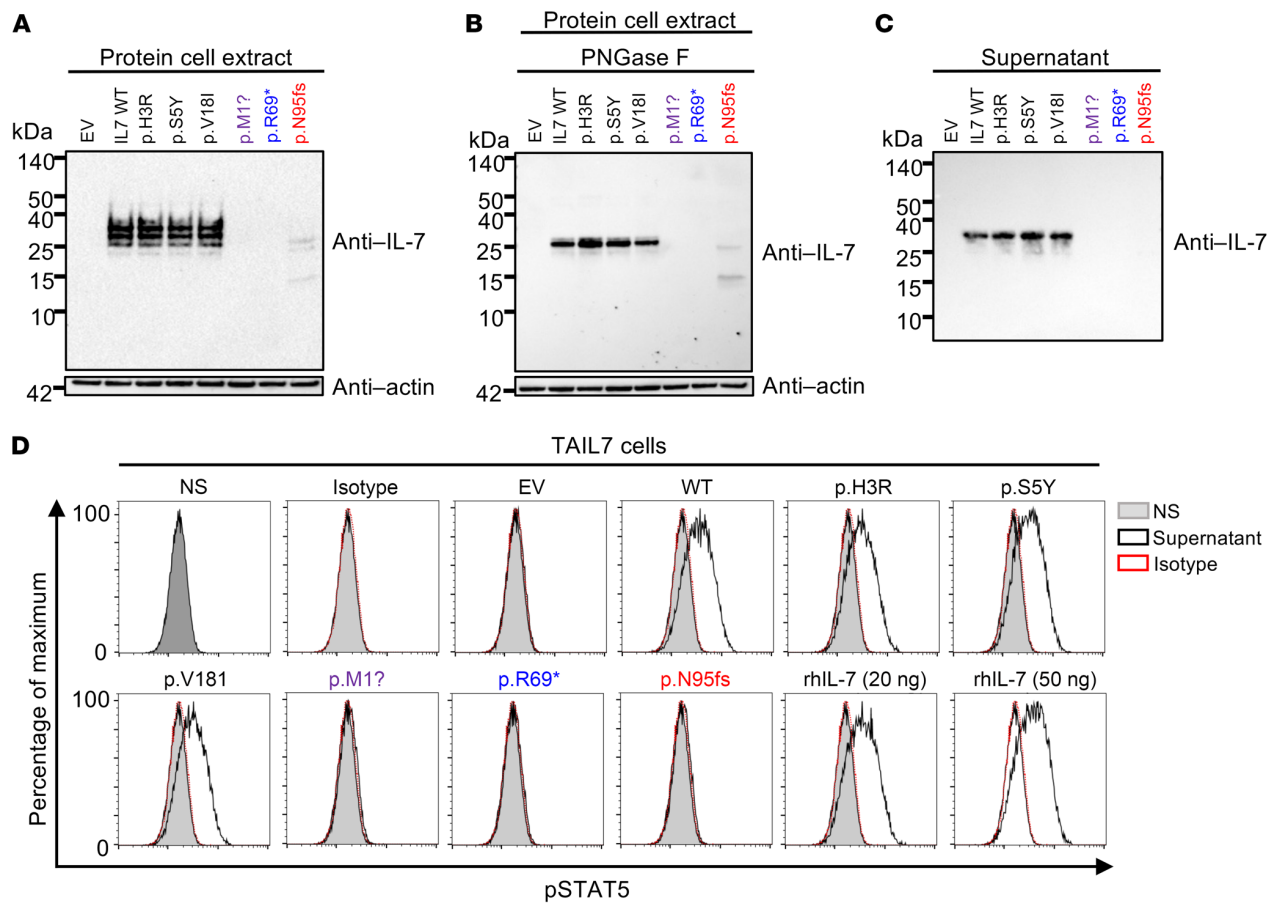
The *IL7* mutant alleles are biochemically deleterious in an overexpression system. (**A**–**C**) Western blot of total cell lysates (**A** and **B**) before (**A**), and after (**B**) PNGase F treatment, and of supernatants (**C**) from HEK293T cells transfected with C-terminally DDK-tagged *IL7* WT, the patients’ or gnomAD *IL7* mutant cDNAs, or with empty vector (EV). The data shown are representative of 2 independent experiments. (**D**) STAT5 phosphorylation in TAIL7 cells after 15 minutes of incubation with the supernatants of HEK293T cells transfected with C-terminally DDK-tagged *IL7* WT, patients’ or gnomAD *IL7* mutant cDNAs or with empty vector (EV), or with human recombinant IL-7 (rhIL-7, 20 or 50 ng/mL). NS, no supernatant (gray); supernatant (black line); and isotype (red dashed line). Representative data from 2 independent experiments are shown.

The IL-7 protein contains 177 amino acids; it has a signal sequence of 25 amino acids and a calculated molecular weight (MW) of 17.4 kDa (31) (Figure 1D). We transiently transfected HEK293T cells with an empty pCMV6 C-terminally DDK-tagged expression vector (EV) or the same vector containing the WT, patients’ (c.3G>A, c.205A>T, and c.284del), or biallelic gnomAD (c.8A>G, c.14C>A, and c.52G>A) *IL7* alleles. All alleles generated similar amounts of *IL7* mRNA (Supplemental Figure 2A). Western blotting of total cell extracts with a monoclonal antibody (mAb) directed against the N-terminal (amino acids 1–100) region of human IL-7, or with a mAb against the C-terminal DDK revealed a protein product with an apparent MW of 25–35 kDa for the WT and the 3 gnomAD missense variants, whereas the patients’ alleles yielded no (c.3G>A/p.M1? and c.205A>T/p.R69*) protein product or a truncated (~15–25 kDa) protein band of low intensity (c.284del/p.N95fs) (Figure 2A and Supplemental Figure 2B). Following deglycosylation by PNGase F, a 25 kDa protein was detected on Western blots of cell extracts from HEK293T cells transfected with plasmids containing WT *IL7* cDNA or cDNAs corresponding to the 3 *IL7* alleles found in gnomAD v2.1, whereas no band (c.3G>A/p.M1? and c.205A>T/p.R69*) or a weak band corresponding to a smaller, 15 kDa protein (c.284del/p.N95fs) was detected on Western blots of cell extracts from HEK293T cells transfected with plasmids containing cDNAs corresponding to the patients’ *IL7* alleles (Figure 2B and Supplemental Figure 2C). Western blotting of the supernatants of HEK293T cells transfected with WT or gnomAD *IL7* alleles revealed a band at about 30 kDa, whereas no band was detected in the supernatants of HEK293T cells transfected with any of the 3 *IL7* alleles from the patients (Figure 2C). Similarly, ELISA detected up to 60 ng/mL IL-7 secreted into the supernatants of HEK293T cells transfected with the WT or any of the 3 *IL7* alleles found in gnomAD, whereas no IL-7 was detected in the supernatants of HEK293T cells transfected with any of the *IL7* alleles from the patients (Supplemental Figure 2D). These data suggest that the patients’ c.3G>A, c.205A>T, and c.284del *IL7* alleles are loss-of-expression in this overexpression system.

### The patients’ IL7 alleles are LOF.

We then evaluated the function of these variants by assessing STAT5 phosphorylation in 2 IL-7–responsive cell lines — TAIL7 (32) and Ba/F3 cells stably expressing IL-7R (33) — upon stimulation with supernatants from transfected HEK293T cells or recombinant human IL-7 (rhIL-7). Supernatants from HEK293T cells transfected for 24 hours with a cDNA encoding WT IL-7 or any of the 3 (c.8A>G/p.H3R, c.14C>A/p.S5Y, and c.52G>A/p.V18I) gnomAD variants induced STAT5 phosphorylation in TAIL7 (Figure 2D) and Ba/F3 cells (Supplemental Figure 2E), at levels similar to those observed following the stimulation of these cells with rhIL-7. By contrast, supernatants from HEK293T cells transfected with any of the patients’ (c.3G>A/p.M1?, c.205A>T/p.R69*, or c.284del/p.N95fs) *IL7* alleles did not induce STAT5 phosphorylation in TAIL7 or Ba/F3 cells (Figure 2D and Supplemental Figure 2E). The patients’ *IL7* alleles are, therefore, LOF in this in vitro system.

### The 6 patients have AR complete IL-7 deficiency.

**Figure 3:**
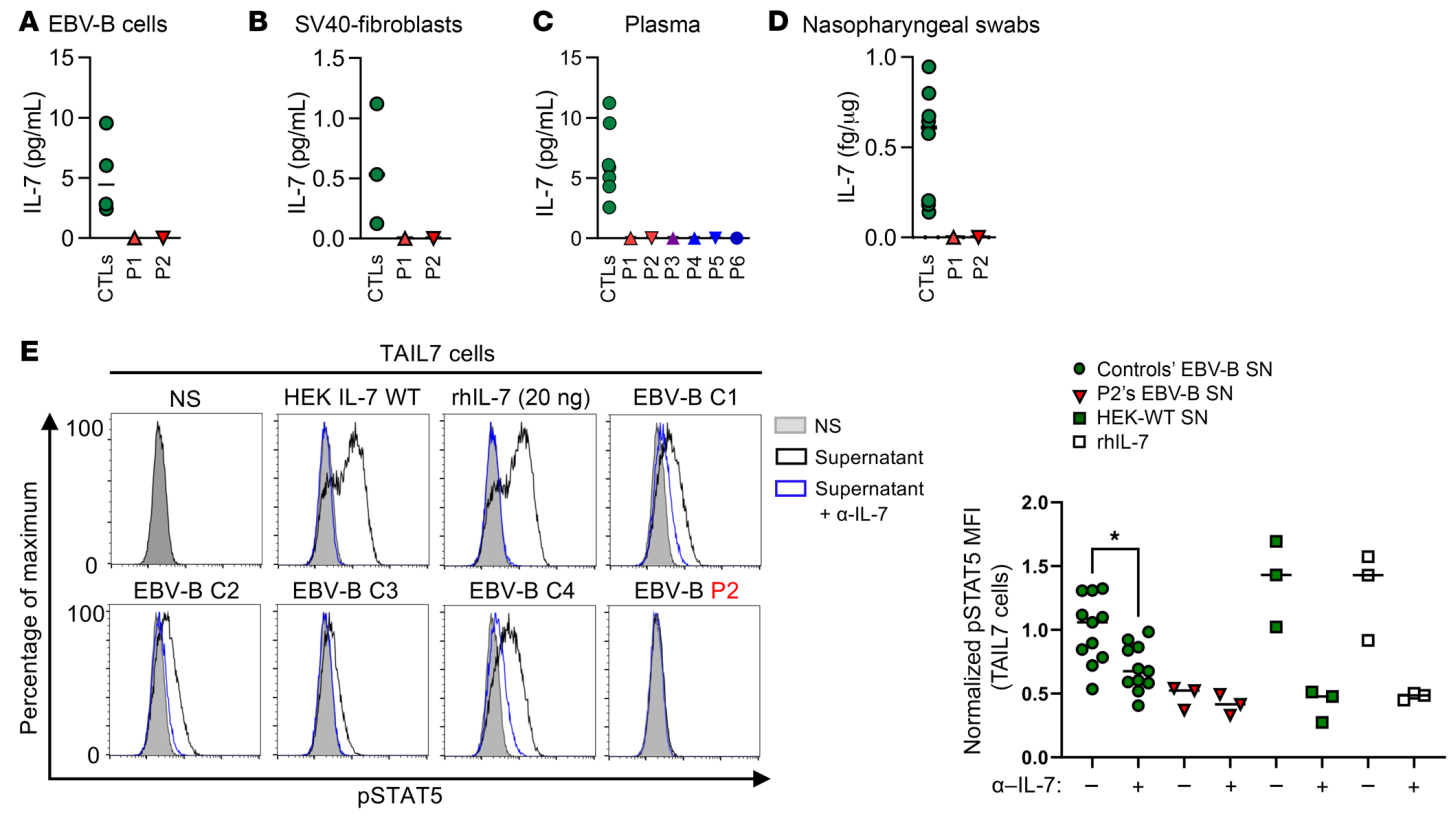
Lack of endogenous IL-7 detection. (**A**–**D**) IL-7 expression, as measured by ELISA on EBV-B cell supernatants (**A**), SV40-fibroblast supernatants (**B**), plasma (**C**), and nasopharyngeal swabs (**D**) from healthy donors (green circles), and the patients. Representative data from 2 independent experiments are shown. CTLs, controls. (**E**) STAT5 phosphorylation in TAIL7 cells after 15 minutes of incubation with supernatants from HEK293T cells transfected with *IL7* WT cDNA, resting EBV-B cells from healthy controls (C1–C4) and P2, or rhIL-7, in the absence or the presence of blocking anti–IL-7 antibody. Left panel shows representative data from 3 independent experiments; right panel represents the quantification of p-STAT5 MFI normalized to the average MFI from 4 healthy controls. Nonparametric Mann-Whitney tests were used for analysis. *P < 0.05. NS, no supernatant (gray); supernatant (black line); and supernatant plus blocking anti–IL-7 antibody (α-IL-7, blue line). SN, supernatant.

Seven human *IL7* transcripts — the full length/canonical and 6 alternative splicing transcripts — have been reported (34) (Supplemental Figure 3A). However, recent tissue-specific bulk RNA-seq data suggest that *IL7* mRNA is strictly limited to the canonical transcript in all tissues in which it is detectable (GTEx portal, https://www.gtexportal.org/home/gene/IL7). We used nonhematopoietic (simian virus 40 [SV40]–immortalized fibroblasts) and hematopoietic (Epstein-Barr virus–immortalized B [EBV-B] cells) cells to assess the impact of the *IL7* variant of P1 and P2 (c.284del) on endogenous *IL7* mRNA and IL-7 protein expression. RT-qPCR analysis revealed similar levels of *IL7* mRNA in both EBV-B cells and SV40-fibroblasts from healthy controls, P1, and P2 (Supplemental Figure 3, B and C). In addition to the canonical transcript, *IL7* mRNA full-length PCR analysis revealed the presence of 3 other transcripts in EBV-B cells and SV40-fibroblasts of healthy controls, P1, and P2 (Supplemental Figure 3, D and E). Sanger sequencing of the TOPO-TA–cloned PCR products showed that more than 40% of the clones contained the canonical *IL7* transcript, in both healthy donors and patients’ cells, and that clones containing 3 other *IL7* transcripts were detected in similar proportions among donors’ and patients’ EBV-B cells. (Supplemental Figure 3F). The IL-7 protein is produced principally by stromal and epithelial cells in lymphoid organs (bone marrow, thymus, lymph nodes, and tonsils), lungs, and skin (24, 35–39). Using a high-sensitivity ELISA kit, we were unable to detect IL-7 in the supernatants of resting EBV-B cells or SV40-fibroblasts derived from P1 and P2, contrasting with our findings for the supernatants of healthy control cells (Figure 3, A and B). Furthermore, no IL-7 was detectable in plasma samples from any of the patients (P1–P6), or in nasopharyngeal samples from P1 or P2, whereas IL-7 was detected in the corresponding samples from healthy donors (Figure 3, C and D). All the other cytokines tested were detected in plasma or nasopharyngeal samples from P1 and P2, at levels similar to those for healthy donors (Supplemental Figure 3, G and H). Finally, we assessed STAT5 phosphorylation in TAIL7 cells incubated with supernatants from EBV-B cells from P2 and healthy controls. Supernatants from controls’ EBV-B cells or HEK293T cells transfected with WT *IL7* cDNA induced STAT5 phosphorylation at levels similar to those observed following stimulation with rhIL-7 (Figure 3E). In contrast, no STAT5 phosphorylation was observed in TAIL7 cells incubated with P1’s EBV-B cell supernatants, as observed for supernatants from controls’ EBV-B cells treated with an IL-7–neutralizing antibody (Figure 3E). Overall, these results suggest that the 6 patients have AR complete IL-7 deficiency.

### Impaired development of specific T lymphocyte subsets in patients with AR IL-7 deficiency.

**Figure 4:**
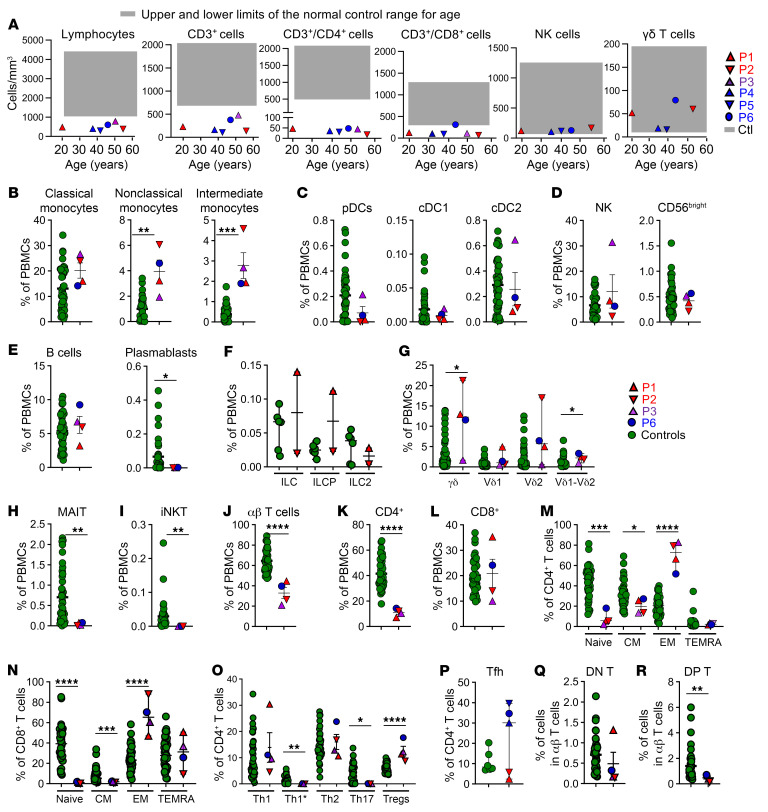
Impaired development of specific T lymphocyte subsets. (**A**) Counts of lymphocytes, CD3^+^, CD3^+^CD4^+^, CD3^+^CD8^+^, NK, and γδ T cells in the blood of the 6 (P1–P6) IL-7–deficient patients. The upper and lower limits of the normal range for age are indicated by gray boxes. (**B**–**R**) Frequencies of several leukocyte subsets in IL-7–deficient patients (P1–P3 and P6) and age-matched healthy controls (green dots), as assessed by spectral flow cytometry on cryopreserved PBMCs, (**B**) classical monocytes (CD14^+^CD16^−^), nonclassical monocytes (CD14^dim^CD16^+^), and intermediate monocytes (CD14^+^CD16^+^), (**C**) plasmacytoid dendritic cells (pDCs) (Lin^–^HLA-DR^+^CD11c^–^CD123^+^), conventional DC type 1 (cDC1) (Lin^–^HLA-DR^+^CD11c^+^CD1c^+^CD141^–^) and cDC2 (Lin^–^HLA-DR^+^CD11c^+^CD1c^–^CD141^+^), (**D**) NK and NK CD56^bright^ cells, (**E**) B cells and plasmablasts, (**F**) innate lymphoid cells (total ILCs, ILCPs [CD117^+^CRTh2^–^], and ILC2s [CRTh2^+^]), (**G**) total TCR-γδ^+^, TCR-γδ1^+^, TCR-γδ2^+^, and TCR-γδ1^+^γδ2^+^ T cells, (**H**) MAIT (MR1^+^TCR-Vα7.2^+^), (**I**) iNKT, (**J**) total α/β T cells, (**K**) α/β CD4^+^ T cells, (**L**) α/β CD8^+^ T cells, among PBMCs, in controls and patients. (**M** and **N**) Frequencies of naive (CD45RA^+^CCR7^+^), central memory (CD45RA^−^CCR7^+^), effector memory (CD45RA^−^CCR7^−^), and TEMRA (CD45RA^+^CCR7^−^) cells among the CD4^+^ (**M**) and CD8^+^ (**N**) T cells. (**O**) Frequencies of the Th1 (CXCR5^−^CXCR3^+^CCR4^−^CCR6^−^), Th1* (CXCR5^−^CXCR3^+^CCR4^−^CCR6^+^), Th2 (CXCR5^−^CXCR3^−^CCR4^+^CCR6^−^), Th17 (CXCR5^−^CXCR3^−^CCR4^+^CCR6^+^), and Treg (CD3^+^CD4^+^CD25^+^CD127^−^) subsets among CD4^+^ T cells in controls and patients. (**P**) Frequencies of Tfh (CXCR5^+^) cells among CD4^+^ T cells in controls and patients. (**Q**–**R**) Frequencies of α/β DN T cells (CD3^+^CD4^−^CD8^−^) (**Q**), and of α/β DP T cells (CD3^+^CD4^+^CD8^+^) (**R**) among the total α/β T cells of controls and patients. Nonparametric Mann-Whitney tests were used for analysis. *P < 0.05, **P < 0.01, ***P < 0.001, ****P < 0.0001.

We investigated the impact of human IL-7 deficiency on leukocyte development by analyzing whole blood from healthy donors and patients. An analysis of blood from the patients showed that counts of polymorphonuclear neutrophils and monocytes (Supplemental Figure 4A), and of γδ T and NK cells were within the normal ranges (Figure 4A). By contrast, total lymphocyte and α/β T cell counts (CD3^+^, CD3^+^CD4^+^, and CD3^+^CD8^+^ T cells) were very low in all patients (29) (Figure 4A, Supplemental Figure 4A, and Supplemental Table 2). We then analyzed cryopreserved peripheral blood mononuclear cells (PBMCs) from healthy donors, P1–P3, and P6 by spectral flow cytometry. We also analyzed whole blood from a 4-month-old SCID patient with complete AR IL-7R deficiency (clinical details provided in Supplemental Methods) by cytometry by time of flight. The IL-7–deficient patients had frequencies of non–T cell leukocyte subsets, including myeloid, NK, and B cells, similar to those in healthy controls. However, the proportions of nonclassical and intermediate monocytes were slightly increased, and those of plasmablasts slightly reduced compared with healthy donors (Figure 4, B–E). The frequencies of innate lymphoid cells (ILCs), ILC progenitors (ILCPs), ILC2 (Figure 4F), and γδ T, Vδ1^+^ and Vδ2^+^ γδ T cells (Figure 4G) were also within the normal ranges in the IL-7–deficient patients. By contrast, the proportions of mucosal-associated invariant T (MAIT) cells, invariant natural killer T (iNKT) cells, total α/β T cells, α/β naive CD4^+^ and CD8^+^ T cells, and central memory T cells were low, whereas the frequencies of CD4^+^ and CD8^+^ effector memory T cells were high, and those of CD4^+^ and CD8^+^ terminally differentiated T cells were within the normal range (Figure 4, H–N). Among CD4^+^ T cells, the frequencies of Th1, Th2, and Tfh cells were within the normal ranges, while those of Th1* (CXCR5^−^CXCR3^+^CCR4^−^CCR6^+^) and Th17 cells were low and those of Tregs were high (Figure 4, O and P). Finally, the frequencies of α/β TCR DN T cells were within the normal range, while those of α/β TCR DP T cells were low in the IL-7–deficient patients (Figure 4, Q and R). The IL-7R–deficient patient tested had frequencies of the monocyte, plasmacytoid DC (pDC), and NK cell subsets within the control range, and of B cells surprisingly in the low range of the controls (Supplemental Figure 4, B–E), whereas the γδ T, MAIT, iNKT, α/β T, CD4^+^ T, CD8^+^ T, and α/β DN T cell subsets were all undetectable (Supplemental Figure 4, F–L). Collectively, these results suggest that inherited IL-7 deficiency impedes the development of MAIT, iNKT, and α/β T cells, with naive and central memory CD4^+^ and CD8^+^ T cells, Th1*, and Th17 cells particularly affected, while sparing other lymphoid and myeloid leukocyte subsets.

### Aberrant transcriptional profiles in IL-7–deficient leukocytes at baseline.

**Figure 5:**
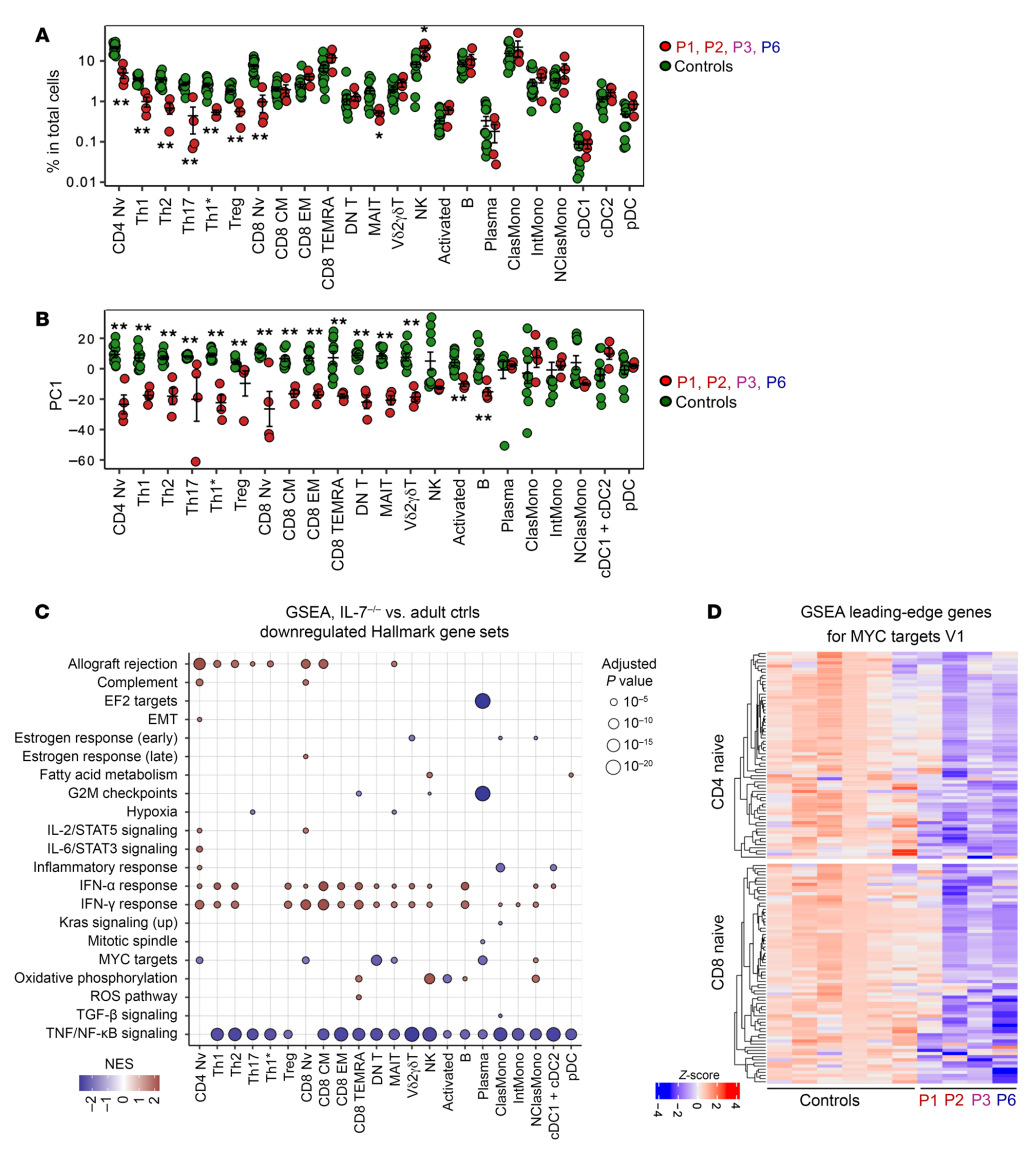
Single-cell transcriptomic analysis. Cryopreserved PBMCs from P1–P3 and P6, together with cryopreserved PBMCs from 2 healthy adult controls, were analyzed. The data were integrated with historical controls via Harmony (40). (**A**) Relative abundance of each leukocyte subset identified through clustering analysis. (**B**) Batch-corrected principal component analysis (PCA). The first principal components (PC1, *y* axis) are shown for each individual leukocyte subset. (**C**) Gene set enrichment analysis (GSEA). Genes were ranked based on the fold-change differences between patients and healthy controls estimated by pseudobulk differential expression analysis. Gene ranks were projected against the Hallmark gene sets (https://www.gsea-msigdb.org/gsea/msigdb/genesets.jsp?collection=H). Selected gene sets related to immune responses are shown. (**D**) GSEA for MYC target V1 genes in naive CD4^+^ and CD8^+^ T cells from IL-7–deficient patients (P1–P3 and P6), and 6 adult controls. The heatmap shows adjusted *z* score values for each sample as a color gradient from blue for transcripts that were detected but below our significance cutoff values (downregulated), through purple, to red for adjusted *z* score values above our significance cutoff values (upregulated). Nonparametric Mann-Whitney tests were used for analysis. *P < 0.05, **P < 0.01.

We further investigated the development and phenotype of leukocyte subsets in P1–P3 and P6 by performing single-cell RNA-seq (scRNA-seq) on cryopreserved PBMCs, together with cryopreserved PBMCs from 2 healthy donors matched for age and sex. Historical healthy controls were also included via Harmony (40). Two-round sequential clustering analysis identified 23 leukocyte subsets (Supplemental Figure 5A) (41). Consistent with the flow cytometry immunophenotyping results, the patients had significantly lower proportions of naive CD4^+^ and CD8^+^ T cells, Th17, and Th1* cells, but also of Th1 and Th2 cells and Tregs. MAIT cell levels were slightly but significantly lower than those in healthy controls, whereas the proportion of NK cells was moderately higher. The frequencies of other leukocyte subsets, such as memory CD8^+^ T cells, Vδ2^+^ γδ T cells, B cells, plasmablasts, and myeloid cells (classical/intermediate/nonclassical monocytes, conventional DCs type 1 and 2, and pDCs) were within the normal ranges (Figure 5A). Pseudobulk principal component analysis (PCA) revealed that the patients’ T lymphocytes (naive CD4^+^, Th1, Th2, Th17, Th1*, Tregs, CD8^+^, MAIT, and Vδ2^+^ γδ T cells) and NK cells had altered transcriptional profiles relative to healthy controls (Figure 5B). PCA also revealed different transcriptional profiles in the patients’ total B cells, despite the apparently normal proportions of these cells in the blood (Figure 5B). Gene set enrichment analysis (GSEA) revealed a downregulation of genes involved in NF-κB signaling relative to healthy controls across the lymphoid and myeloid leukocyte subsets of the patients (Figure 5C). Moreover, the patients’ naive CD4^+^ and CD8^+^ T cells displayed a reduced expression of c-Myc–driven genes (Figure 5, C and D), suggesting that the few naive T cells that develop in the absence of IL-7 have impaired proliferative capacities. Collectively, these results suggest that inherited IL-7 deficiency alters the transcriptional profiles of both T and B cells, and to some extent that of NK cells, with a marked impairment in expression of c-Myc–driven genes in naive CD4^+^ and CD8^+^ T cells.

### Impaired early T cell development in patients with AR IL-7 deficiency.

**Figure 6:**
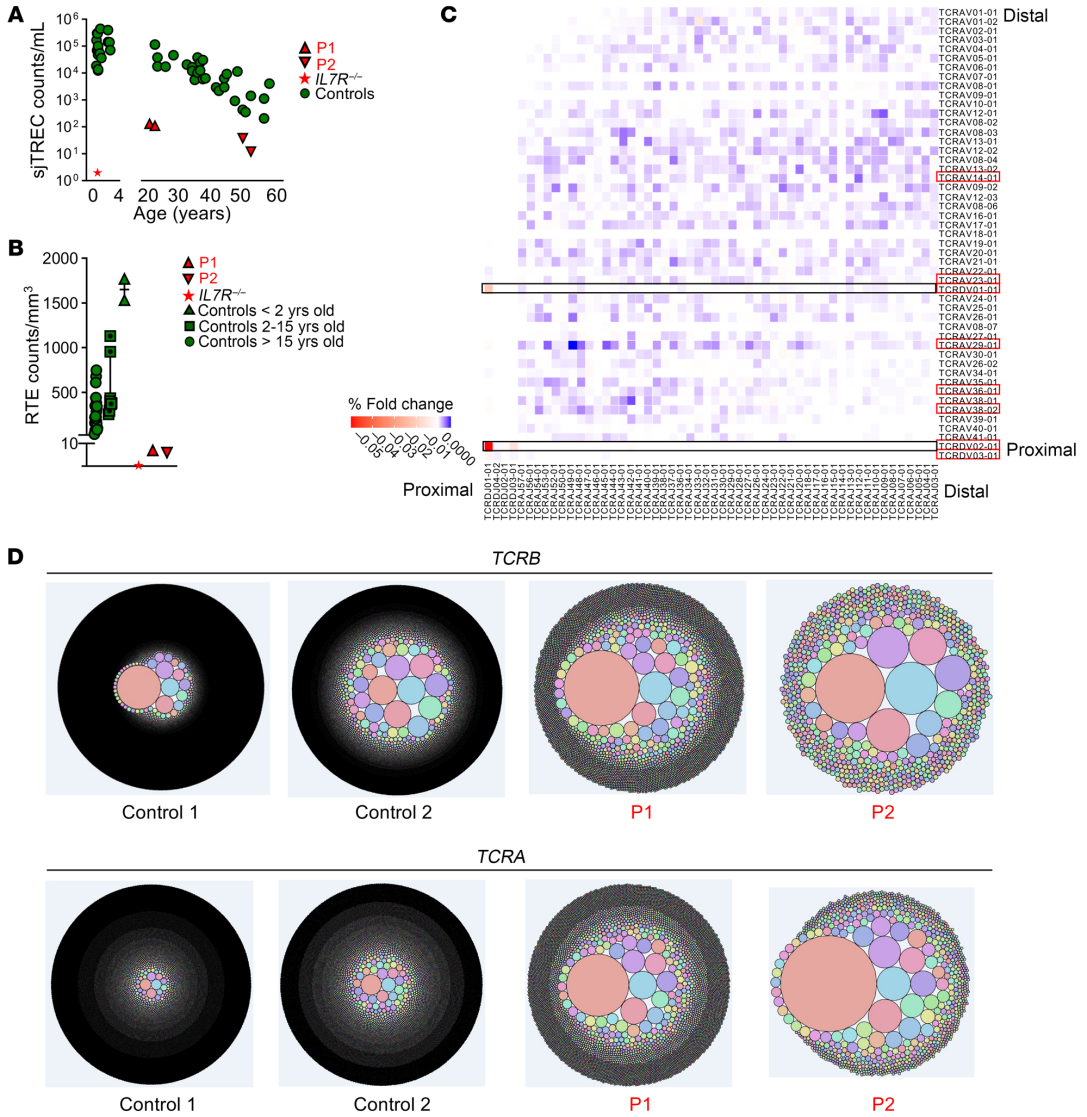
Impaired early T cell development in AR IL-7 deficiency. (**A**) TCR excision circles (TRECs) in P1 (at 20 and 22 years of age), P2 (at 54 and 57 years of age), and an IL-7R–deficient patient (at 4 months of age), relative to healthy age-matched controls. The sjTREC count in blood (sjTREC/mL) is presented as a function of age. (**B**) Counts of recent thymic emigrant (RTE) cells, defined as CD3^+^CD4^+^CD45RA^+^CD31^+^ cells in fresh blood from P1, P2, an IL-7R–deficient patient, and age-matched healthy controls. (**C**) Heatmap of paired gene rearrangements of the *TRAD* locus in whole-blood samples from P1, P2, and 2 age-matched controls. The fold-change differences are indicated below. The red color highlights V-J gene pairings overused in patients and the blue color highlights V-J gene pairings overused in controls. The *TRAV* and *TRDV* genes known to be involved in TCR-δ rearrangements are indicated with a red rectangle. The TCR-δ1 (TRDV1:TRDJ1) rearrangement is indicated with a black rectangle. (**D**) *TCRB* and *TCRA* rearrangements, represented by Treemap plots, for whole-blood samples from P1, P2, and 2 age-matched healthy controls.

Given the low proportions of naive T cells in all the IL-7–deficient patients tested, and the role of IL-7/IL-7R signaling in human T cell development (24, 35, 42, 43), we evaluated thymic T cell output. A computed tomography scan showed soft tissue attenuation consistent with a remnant thymus for P1 (21 years old) and, as expected, no thymic tissue for P2 or P3, who both were 56 years old at the time of sampling (Supplemental Figure 6A). We evaluated TREC levels by quantitative nested PCR on whole blood from P1, P2, and the IL-7R–deficient patient, as previously described (44, 45). Both P1 and P2 had much lower signal-joint TREC (sjTREC) counts than age-matched healthy individuals, suggesting that the thymus was able to produce some T cells, whereas the IL-7R–deficient patient had almost no sjTRECs, consistent with an almost complete loss of thymopoiesis (Figure 6A). We then quantified recent thymic emigrant (RTE) cells, the T cell subsets that had recently completed intrathymic development and exit from the thymus to the periphery (46). We detected very low absolute counts of RTE (CD3^+^CD4^+^CD45RA^+^CD31^+^) cells in both P1 and P2, but with normal proportions of these cells among CD4^+^ T cells, whereas no RTE cells were detectable in the IL-7R–deficient patient (Figure 6B and Supplemental Figure 6B). These data suggest that thymopoiesis is abolished in AR IL-7R deficiency, but reduced to residual levels in AR IL-7 deficiency.

### Abnormal TCR αβ repertoire in patients with AR IL-7 deficiency.

The impaired thymic T cell development observed in patients with AR IL-7 deficiency and the regulated expression of *IL7R* during T cell thymopoiesis (24) raised the question of whether rearrangements are biased or limited in diversity in the absence of IL-7. We first assessed TCR-β and TCR-α repertoires in DNA extracted from whole-blood samples from P1 and P2, by performing high-throughput sequencing of *TCRB* and *TCRA*. We found no major difference between the patients and controls in terms of *TCRA* and *TCRB* V gene usage, with a normal capacity of the patients to use distal *TCRA* segments (Supplemental Figure 6C). By contrast, a heatmap of paired gene rearrangements at the *TRAD* locus suggested higher frequency and preferential usage of TCRDV01:01-TCRDJ01:01 and TCRDV02:01-TCRDJ01:01 productive V-J rearrangements at the *TCRD* locus (Figure 6C). The amino acid sequence lengths of the CDR3 corresponding to the *TCRAD* and *TCRB* loci appeared normal in both P1 and P2 (Supplemental Figure 6D). However, for both *TCRA* and *TCRB*, we observed a lower productive entropy and higher productive clonality in P1 and P2 than in controls, consistent with an overall decrease in diversity and increase in clonality, respectively (Figure 6D and Supplemental Figure 6E). Collectively, these data suggest that the IL-7–deficient patients have a much less diverse and more oligoclonal TCR-αβ repertoire than healthy individuals. This outcome likely stems from their compromised T cell development, leading to a distinctive peripheral T cell phenotype, notably marked by the low proportions of naive T cells. Nevertheless, some αβ T cells were able to exit the thymus, with a fairly broad usage of the *TCRA* and *TCRB* V genes.

### Impaired peripheral T lymphocyte function in patients with AR IL-7 deficiency.

**Figure 7:**
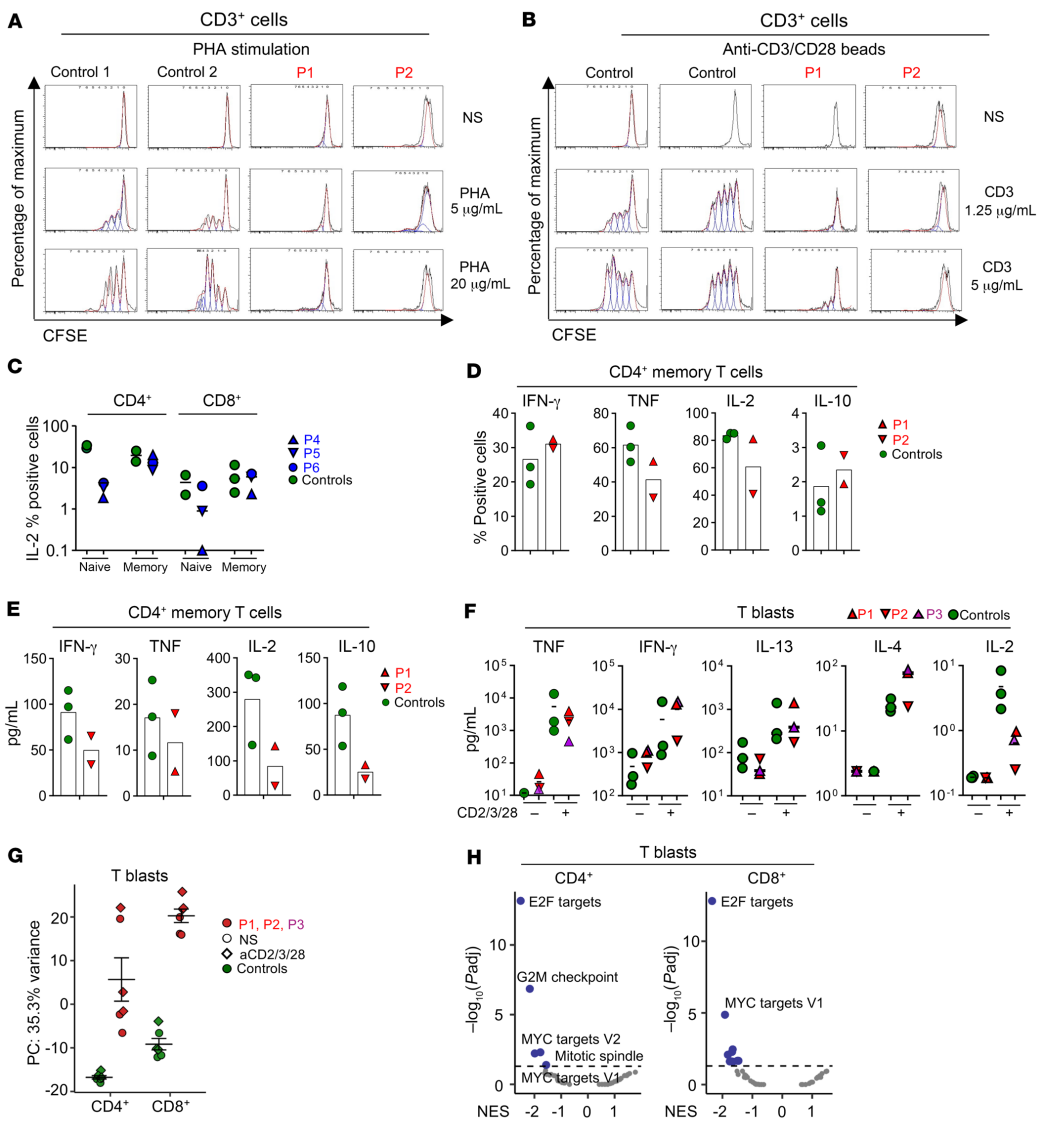
Peripheral T cell functions. (**A** and **B**) Fresh PBMCs from age-matched controls (only 2 controls are shown), P1, and P2 were incubated for 4 days with (**A**) PHA (5 μg/mL or 20 μg/mL), or (**B**) with beads coated with anti-CD3 and anti-CD28 mAbs (anti-CD3 mAbs were used at concentrations of 1.25 μg/mL and 5 μg/mL). Histograms show CFSE dilution for CD3^+^ T lymphocytes. Representative results from 2 independent experiments are shown. (**C**) Intracellular IL-2 production by CD4^+^ or CD8^+^ naive or memory T cells after stimulation with PMA plus ionomycin for 4 hours and flow cytometry analysis, for IL-7–deficient patients (P4, P5, and P6) and 3 healthy controls. (**D** and **E**) Memory CD4^+^ T cells from healthy controls, P1, and P2 were stimulated with PMA plus ionomycin after 5 days of culture; the percentages of cells expressing IFN-γ, TNF, IL-2, and IL-10 intracellularly were determined by flow cytometry (**D**) and secreted cytokines were assessed by LEGENDplex (**E**). (**F**) T cell blasts from P1–P3 and 3 healthy controls were stimulated for 2 hours with an anti-CD2/anti-CD3/anti-CD28 mAb cocktail. Secreted cytokines were determined by LEGENDplex. (**G** and **H**) Bulk RNA transcriptomic analysis on sorted CD4^+^ and CD8^+^ T cell blasts from P1–P3 and 3 healthy adult controls. Principal component analysis (PCA) results are shown for sorted CD4^+^ and CD8^+^ T cell blasts in the absence of stimulation or after stimulation with the anti-CD2/anti-CD3/anti-CD28 mAb cocktail (**G**). Normalized enrichment score (NES) analyses are shown for unstimulated sorted CD4^+^ and CD8^+^ T cell blasts (**H**).

We then investigated the impact of IL-7 deficiency on T cell function. Polyclonal stimulation of fresh PBMCs from P1 and P2 with phytohemagglutinin (PHA) or beads coated with mAbs against CD3 and CD28 (anti-CD3/anti-CD28) for 4 days resulted in almost no CD3^+^ T cell proliferation relative to healthy control cells, on CFSE dilution (Figure 7, A and B, and Supplemental Figure 7A). Furthermore, no T cell proliferation was observed after the stimulation of fresh PBMCs from P2 with tetanus toxoid and candidin (Supplemental Figure 7, B and C). This impairment of T cell proliferation may be due to the low naive T cell proportions in peripheral PBMCs (Figure 4, M and N), and to their lower levels of IL-2 production, as observed in naive CD4^+^ and, to a lesser extent, naive CD8^+^ T cells from P4–P6 (Figure 7C). After 5 days under Th0 conditions, the memory CD4^+^ T cells of P1 and P2 displayed cytokine intracellular induction and secretion within or in the lower ranges of the healthy controls (Figure 7, D and E, and Supplemental Figure 7, D and E). We were able to expand a few T cell blasts from P1, P2, and P3 with anti-CD2/anti-CD3/anti-CD28 mAbs. These cells had lower levels of IL-2 production, whereas other cytokines (TNF, IFN-γ, IL-4, and IL-13) were produced in amounts similar to those for healthy donors (Figure 7F and Supplemental Figure 7F). An analysis of RNA-seq data for sorted CD4^+^ and CD8^+^ T cell blasts from P1–P3 revealed an aberrant transcriptomic profile in both subsets, regardless of restimulation (Figure 7G), and low TCR repertoire diversity (Supplemental Figure 7G). GSEA revealed a marked downregulation of E2F and MYC target genes in both CD4^+^ and CD8^+^ T cell blasts, and of G_2_/M checkpoint–related genes in CD4^+^ T cell blasts (Figure 7H and Supplemental Figure 7H). Collectively, these data suggest that IL-7 deficiency impedes the proliferation of peripheral T lymphocytes without markedly altering their ability to produce a broad range of cytokines.

### Preserved B cell numbers and function in patients with AR IL-7 deficiency.

IL-7/IL-7R signaling has been shown to play a crucial role in B cell development in mice, especially by promoting the progression from the pro–B to the pre–B cell stage (47–49). A recent study suggested that human IL-7R signaling promotes early B cell progenitor proliferation, differentiation, and expansion (25). However, patients with AR IL-7R deficiency have subnormal to normal B cell counts (15, 17). We therefore evaluated the B cell compartment and humoral responses of IL-7–deficient patients. B cell counts were within or slightly below the range for healthy controls, with normal frequencies of total B cells, transitional, naive, memory, and switched memory B cells (Figure 4E, Supplemental Figure 4A, and Supplemental Figure 8A), whereas the IL-7R–deficient patient tested had normal levels of transitional and naive B cells, but no memory and switched memory B cells (Supplemental Figure 8B). Serum IgG, IgA, IgM, and IgE levels were also within the ranges of healthy controls (Supplemental Figure 8C). Furthermore, the patients had detectable serum antibodies (Abs) against pneumococcal polysaccharides and protein antigens (Supplemental Table 3). We assessed B cell function in vitro by measuring Ig secretion after B cell stimulation with CD40L alone or in combination with CpG or IL-21. Naive and memory B cells from both P1 and P2 produced amounts of IgG, IgA, and IgM upon stimulation similar to those for naive and memory B cells from healthy controls (Supplemental Figure 8D). We assessed the Abs directed against a wide array of microbial species present in the serum of P1–P4 by performing VirScan-phage immunoprecipitation–sequencing (PhIP-seq). All 4 patients were able to mount detectable humoral responses to various microbes, reflecting their previous exposure to a wide range of common pathogens (Supplemental Figure 8E). Clinical autoimmunity was not a common feature of the patients, but we investigated the possible presence of subclinical autoimmunity. We detected no classical autoimmune markers, such as Abs against soluble nuclear antigens (anti-ENAs), anti-neutrophil cytoplasm Abs (ANCAs), or anti-nuclear Abs (ANAs), on ELISA and indirect immunofluorescence assays on plasma samples from P1 or P2 (data not shown). We also assessed the auto-Ab repertoire in plasma samples from P1 and P2 with HuProt, a microarray assay capable of detecting auto-Abs against 20,000 unique full-length human proteins (50). Only a very small number of overlapping autoantigens were detected in the plasma samples from P1 and P2, similar to those found in plasma samples from some healthy controls (Supplemental Figure 8F). Overall, these data suggest that IL-7 deficiency has a mild effect on total B cell counts without profoundly impairing the development, differentiation, or function of these cells.

## Discussion

We describe 6 adult patients with AR complete IL-7 deficiency and CID. The myeloid and NK cell compartments were essentially intact, as in IL-7R–deficient patients (this study and refs. 15, 17), further confirming that IL-7/IL-7R signaling is not essential for the development of these cells. The B cell compartment also remained largely unaltered, with normal proportions of memory and switched memory B cells, diverging from the IL-7R–deficient patient who lacks these cells possibly secondary to the T cell deficiency (this study). In marked contrast, the IL-7–deficient patients displayed severe αβ T cell lymphopenia, whereas their γδ T cell counts and proportions were, surprisingly, similar to those in healthy individuals. This finding differs from that for IL-7R–deficient patients who entirely lack or have very low levels of both αβ and γδ T cells (this study and refs. 15, 17). The IL-7–deficient patients had very low counts of TRECs, RTE cells, and naive CD4^+^ T cells, all of which were undetectable in the IL-7R–deficient patient tested, indicating poor inthrathymic proliferation and/or survival, and an impairment, but not total abolition of thymic output. Together, these data demonstrate that IL-7R signaling is indispensable and acts very early in thymopoiesis (pro-T stage) (16, 51), whereas IL-7 deficiency results in a partial impairment, possibly at later stages, during thymic T cell differentiation, affecting the immature SP or DP stage (Supplemental Figure 9).

Innate T cell populations, including MAIT and iNKT cells, also seemed to be affected by the absence of IL-7, as their proportions were at the lower end of the healthy control range, in both IL-7– and IL-7R–deficient patients. By contrast, the proportions of ILC (total ILCs, ILCPs, and ILC2s) in all IL-7–deficient patients tested were similar to those in healthy individuals, while these cells were not evaluated in IL-7R–deficient patients. Among αβ T subsets (for which total counts were low), some subsets, including CD4^+^ (naive, central memory, Th17, and Th1*) and CD8^+^ (naive and central memory) T cells had low proportions, whereas the proportions of others were normal (Th1, Tfh) or slightly high (Tregs) relative to healthy individuals. The residual peripheral αβ T cells, which mostly had an effector memory phenotype, displayed subnormal to normal cytokine production, but proliferated poorly in response to stimulation with mitogens or antigens in vitro. They also had a low TCR repertoire diversity, with increased clonality, at least partly reflecting impaired thymic T cell development, but homeostatic T cell proliferation in the periphery. Despite these T cell defects, CD4^+^ T cells that were generated in the IL-7–deficient patients — most likely Tfh cells — acquired sufficient functionality to induce productive humoral immune responses, evidenced by intact proportions of memory B cells and levels of serum Ig, as well as and protective levels of vaccine- and pathogen-specific IgG. Overall, these observations suggest that IL-7–dependent and IL-7–independent programs operate differently during the development, maintenance, and differentiation of human T cell subsets.

All 6 patients suffered from persistent, disseminated, and treatment-refractory HPV-driven cutaneous warts (a phenotype commonly referred to as recalcitrant warts; ref. 52), probably due to their T cell lymphopenia (52–55) (Supplemental Table 1). Human genetic studies of patients with recalcitrant warts have highlighted the critical importance of T cell–dependent adaptive immunity, as exemplified by AR CD28 deficiency (53, 56, 57). P3 and P4 developed skin squamous cell carcinomas in the context of α-HPV infection, suggesting distinct epidemiological and biological mechanisms of skin carcinogenesis compared with those associated with commensal β-HPVs (which underlie flat warts and skin malignancies in both patients with epidermodysplasia verruciformis and in the general population) (58, 59). Additionally, 4 patients (P1, P2, P4, and P5) also developed invasive fungal diseases, notably cryptococcal meningitis (P2, P4, and P5). Autoantibodies neutralizing GM-CSF, which can underlie cryptococcal meningitis (60, 61), were not detected in their plasma (data not shown). Cryptococcal meningitis remains a major cause of meningitis in HIV-infected individuals, accounting for over 65% of such cases (62). Similarly, cryptococcosis remains as one of the most prevalent infection in patients with idiopathic CD4^+^ T cell lymphopenia (55). Only P1 and P2 suffered from mycobacterial diseases (*Mycobacterium* *tuberculosis* and *Mycobacterium spp*., respectively), potentially due to impaired MAIT- and CD4^+^ T cell–mediated IFN-γ production (63, 64). Recurrent VZV herpes infections were reported in P2 and P4; P2 had autoantibodies neutralizing a low concentration (100 pg/mL) of IFN-α, which can underlie infections with HSV-1 and VZV (65–67), while P4 was not tested (data not shown). The patients’ CD4^+^ T cell lymphopenia, possibly coupled with reduced resident memory CD4^+^ T cells, may explain these infections. Except for a recent diagnosis of antiphospholipid syndrome in P3, none of the patients displayed clinical signs of autoimmunity, which may be attributed to their compromised yet functional thymic output and normal or increased proportions of peripheral Tregs. Overall, the clinical phenotypes observed in patients with AR IL-7 deficiency closely resemble those in individuals with idiopathic CD4^+^ T cell lymphopenia (55).

Mice deficient in either IL-7 or IL-7R show a strong impairment of αβ T cell development within the thymus, with IL-7R deficiency having a more pronounced impact (47, 48) (Supplemental Table 4). Specifically, *Il7r*^–/–^ mice display an early block in thymopoiesis, transitioning from CD44^+^CD25^–^ pre-pro-T cells to CD44^+^CD25^+^ pro-T cells, while *Il7^–/–^* mice show a blockade at a later stage, from CD44^+^CD25^+^ pro-T cells to CD44^–^CD25^+^ pre-T cells (47, 68–70). In the periphery, both *Il7^–/–^* and *Il7r^–/–^* mice show a profound reduction in lymphocyte numbers, particularly marked by low levels of αβ T cells and an absence of γδ T cells (69, 71). In contrast with humans with AR IL-7 or IL-7R deficiency, who maintain normal peripheral B cell counts, both *Il7^–/–^* and *Il7r^–/–^* mice exhibit markedly reduced B cell numbers, due to impaired development in the bone marrow from pro-B to pre-B and pre-pro B to pro-B stages, respectively (47, 48, 72). Both *Il7^–/–^* and *Il7r^–/–^* mice maintain NK cell counts comparable to those of WT mice, similar to humans harboring genetic defects in the same genes (71). Mice deficient for *Tslp* or *Crlf2* have normal counts of T, B, and NK cells (72–76) (Supplemental Table 4). Overall, these data underscore the specific requirement of IL-7/IL-7R for T and B lymphocyte development in mice, whereas in humans, IL-7R is crucial for T cell development, while IL-7 specifically supports the development of only certain T lymphocyte subsets.

The dissection of the IL-7–dependent and IL-7–independent T cell developmental programs has important clinical implications. IL-7–deficient patients, unlike IL-7R–deficient patients, will not benefit from allogenic hematopoietic stem cell transplantation, thus necessitating alternative treatment strategies. IL-7–deficient patients may benefit from replacement therapy with recombinant IL-7 (77). The addition of IL-7 during the in vitro preparation of T cells for T cell–based immunotherapy has been proposed as a means of improving the effector functions of these cells following autologous transplantation into patients (78). Further investigations of the IL-7–independent T cell development program may reveal human-specific, potentially targetable pathways for improving T cell functionality. The best candidate molecule for targeting would be TSLP, which also signals through IL-7R, together with CRLF2 (27, 28). However, the role of TSLP in humans has not yet been defined, as no TSLP- or CRLF2-deficient individuals have ever been reported. In conclusion, these experiments demonstrate that human IL-7 deficiency is milder than IL-7R deficiency, affecting a limited range of conventional αβ T lymphocyte subsets, and possibly some nonconventional T cells, such as iNKT or MAIT cells. This observation suggests that other IL-7R–binding cytokines, such as TSLP, may govern the IL-7–independent development of T cell subsets in humans.

## Methods

Further information can be found in Supplemental Methods.

### Sex as a biological variable.

We enrolled 7 individuals in our study, 4 women and 3 men. No sex bias has been reported; therefore, it was not considered as a biological variable.

### Statistics.

The statistical analyses for Figure 3E, Figure 4, B–R, and Figure 5, A and B were performed with GraphPad Prism 8.4.3. Nonparametric Mann-Whitney tests were used for analysis. A *P* value of less than 0.05 was considered statistically significant, with *P < 0.05, **P < 0.01, ***P < 0.001, and ****P < 0.0001.

### Study approval and ethics committees.

Informed consent was obtained from all the families according to protocols approved by local IRBs and human research. This study was conducted according to the “Scientific Standards for Technical and Administrative Health Research” established by the Colombian Ministry of Health Resolution 008430 of 1993 and approved by the local review board of the Universidad de Antioquia (F8790-07-0010) and Necker Hospital for Sick Children, France.

### Data availability.

scRNA-seq, RNA-seq data, *TCRA*, and *TCRB* sequencing generated from patients and controls are available in the SRA database (https://www.ncbi.nlm.nih.gov/bioproject/PRJNA1123208 ID: PRJNA1123208). Raw data are provided in the supplemental Supporting Data Values file. HEK293T cells were obtained from ATCC. All biological materials, including cell lines or immortalized cell lines from patients, are available upon request to the corresponding authors under a Material/Data Transfer Agreement.

## Author contributions

CAAF, MO, SU, OMD, AY, J Bohlen, AF, JPS, JR, TLV, QP, KJP, AGD, LVEB, AC, PG, JMD, BCDM, JAA, DMA, LPZ, MMV, MJ, BP, TN, CSM, TK, DR, JLP, NM, SBD, DK, JTB, NL, LA, AL, VB, EJ, J Bustamante, JPDS, SGT, LDN, RC, AAA, and AP performed or supervised experiments, generated and analyzed data, and contributed to the manuscript by providing figures and tables. YS, JFA, FC, M Chaldebas, PZ, and BB performed or supervised computational analyses of data. JTB provided BAF3 and TAIL7 cell lines. JCO, MMVL, AFZV, M Chbihi, LACL, AGD, LH, AL, EVE, PJP, CJM, JAL, YC, RI, VMP, BN, KN, JLF, and KW evaluated and recruited patients. CAAF, MO, J Bohlen, KW, JLC, and AP wrote the manuscript. JLC and AP supervised the project. All authors edited the manuscript.

## Supplementary Material

Supplemental data

Unedited blot and gel images

Supporting data values

## Version 1 10/01/2024

Electronic publication
