# Supplementary material for: IL-7–dependent and –independent lineages of IL-7R–dependent human T cells
Source: J Clin Invest. 2024 Oct 1;134(19):e180251. doi: 10.1172/JCI180251 (PMC11444196; doi:10.1172/JCI180251)
Supplement: Supplemental data [file jci-134-180251-s157.pdf]

Supplemental Methods

Case reports

Patient 1 (P1) is a 23-year-old male born to first-degree consanguineous Colombian parents, and is the second child of three siblings. During the first year of life, he received all the vaccines from the national immunization program without any adverse events and appeared healthy until age one year when he began presenting intermittent fevers, of unknown cause, which resolved with common antipyretics. At 22 months, P1 presented again with intermittent fever, this time associated with a serosanguinous nasal discharge, which partially improved with antibacterial treatments (Amoxicillin, Clavulanate, and ampicillin). Symptoms persisted, leading to a chronic dry cough and progressive erosion of the nasal septum. A diagnosis of sinusitis due to *Histoplasma capsulatum* was established following a biopsy and cultures of the nasal mucosa, which revealed the presence of *H. capsulatum*. A fiberoptic bronchoscopy indicated mild endobronchitis, suggesting pulmonary histoplasmosis. P1 received Amphotericin B (AmB) for eight days, followed by oral Itraconazole (ITZ) for seven months. Post-treatment cultures were negative for *H. capsulatum.* Although a maxilloethmoidectomy improved his condition, nasal septum deformity progressed despite antifungal therapy. Between 2004 and 2014, P1 underwent eight surgeries, including skin flaps, rib cartilage grafts, and tissue expander placement and removal to correct his nasal deformity, with poor results.

At the age of 7 years, he developed productive cough, intermittent fevers, diaphoresis and myalgias for six months, along with mobile and painless bilateral cervical adenopathies. He had household contact with relatives (aunt and uncle) who had confirmed pulmonary tuberculosis. Bacilloscopies of his sputum and gastric fluid revealed acid-fast bacilli (AFB) leading to a diagnosis of pulmonary tuberculosis. He was treated with daily oral isoniazid (H), rifampicin (R), and pyrazinamide (Z), and ethambutol (E), with partial improvement. Three months into anti-TB therapy, he developed blurred vision, worsening cough, subjective fevers, upper and lower limb stiffness, loss of consciousness, and tonic-clonic seizures with focal onset in left arm and subsequent generalized clinical features. A brain CT scan revealed a contrast-enhancing hypodense lesion with surrounding vasogenic edema in the right frontal lobe, suggesting a tuberculoma. Due to clinical improvement with the anti-TB treatment and the absence of *H. capsulatum* isolation, a diagnosis of tuberculosis was favored, and RHZE treatment was extended to one year. Additionally, he was treated with a 21-day cycle of Ampicillin/Sulbactam for the ethmoid/maxillary pansinusitis and valproic acid for one year to manage seizures.

At age 11 years, the patient began experiencing intermittent and progressive headaches, nausea, vomiting, purulent rhinorrhea, dry cough, hyporexia and weight loss for five months. He had been living with a paternal uncle who was later diagnosed with pulmonary TB. Physical examination revealed low weight and height for age, hyperreflexia, and bilateral papilledema. Bacilloscopies of the sputum were positive. A brain MRI showed nodular hyperintense lesions in the cerebellum and signs of hydrocephalus, and a chest CT scan revealed micronodular infiltrates in a budding tree pattern, suggesting pulmonary TB. Molecular testing identified Rifampicin-resistant *Mycobacterium tuberculosis*. P1 was started on a combination therapy of P+E, Ethionamide (Eto), Moxifloxacin, and Kanamycin. After six months, Moxifloxacin and Kanamycin were suspended, and Amino salicylic Acid (PAS) was initiated and administered

for three months. Finally, therapy with P, E, and Eto was continued for up to 18 months. Simultaneously, antifungal therapy (AmB and ITZ) was administered. However, pathological studies from samples obtained by craniotomy and biopsy of the posterior fossa mass revelated chronic granulomatous inflammation with caseous necrosis, negative for fungi or acid-fast bacilli (AFB).

At the age of 12 years, P1 started to develop warts on his hands and feet. Despite multiple treatments including urea, salicylic acid and 5-fluorouracil, there was little improvement. At age 20, he experienced a new episode of recurrent fevers, diaphoresis, dyspnea, and weight loss, lasting one month. Bronchoalveolar lavage identified *H. capsulatum*, and he was treated with AmB and ITZ. Since early 2023, he has been on ITZ (400 mg/d PO) for a relapse of pulmonary histoplasmosis. His siblings and parents are all healthy (Supplemental Table 1).

Patient 2 (P2) is a 60-year-old female born in 1964 to apparently non-consanguineous parents of Hispanic descent, and is the fifth child of six siblings. Her oldest sister died at the age of six years of an unknown cause. She received all vaccines in the national program (IPV, DPT, BCG, HBV, TD, MMR) without apparent adverse events. She remained healthy until the age of seven years, when she developed painful unilateral vesicles affecting her right hemithorax and part of the upper abdomen, diagnosed as severe herpes zoster. She was treated with topical master formulas and analgesics, eventually the lesions resolved without scars. A few months later, she began to develop warts mainly on sun-exposed areas including limbs and face. Despite several consultations and topical treatments with "natural" creams, keratolytic agents, dinitrobenzene, and even electrocauterization, her condition did not improve, and the lesions progressed slowly. At age 25, a dermatologist performed cryotherapy, which resulted in ischemic necrosis of the left thumb, requiring amputation of the distal phalanx of left thumb and discontinuation of the therapy. In 1997, a skin biopsy identified benign squamous papilloma and common warts. Topical treatment was initiated with dinitrochlorobenzene and later changed to sub-cutaneous recombinant human interferon (rhIFN-α2a, Roferon-A, Roche) at the dose of 3 x 10^6 IU/2 x week in cycles of four weeks for several months without improvement. Then, dinitrochlorobenzene was imitated but later replaced by Imiquimod, followed by diphenylcyclopropenone (diphencyprone, DPCP) from 2011 to 2013, with partial improvement. In 2013, she discontinued all topical treatments for her warts, but experienced an increase in the number and spread of warts.

At age 28, she began experiencing mild recurrent frontal and retroocular headaches, lasting for about two months. The headaches became severe and accompanied by nausea and vomiting, prompting her to visit the emergency room (ER) where she was hospitalized with suspected meningitis. A lumbar puncture was performed. The analysis revealed a transparent LCR with total proteins (138 mg/dL; ref 15-60), low glucose (22 mg/dL; ref 40-80), and lymphocytes of 10/µL (ref 0-2). Gram and AFB staining yielded negative results; however, structures compatible with *Cryptococcus neoformans* were observed. The latex agglutination test for *Cryptococcus* was mildly positive with a titer of 1:128. Tests for antibodies against HIV and HLV-2 (DNAR) by ELISA were negative. Consequently, a diagnosis of meningeal cryptococcosis was established. She initially received intravenous Amphotericin B, with doses increasing from 5mg/d up to 35 mg/d over a period of 9 days, alongside 150 ml/d of i.v. Mannitol. However, she developed hypokalemia requiring boluses of KCL potassium.

Subsequent antifungal therapy included oral fluconazole at 400 mg/d. Two weeks later, AmpB was resumed at the dose of 35 mg/d every other day, continued for a total of two months. At this time, a follow-up lumbar puncture showed a cryptococcal latex agglutination titer of 1:32. Due to intermittent shortage of AmpB, her treatment alternated between AmpB and fluconazole until her discharge. Throughout the year, she experienced two episodes of herpes zoster on the right tight, managed with oral acyclovir (800 mg/d) and analgesics. She remained on prophylactic treatment for several weeks.

At the age of 30 years, she developed a chronic dry cough, fever, nocturnal diaphoresis, hyporexia and insomnia, without weight loss. She was hospitalized in Armenia, where sputum tests were positive for AFB, leading to a diagnosis of pulmonary tuberculosis. She began antimycobacterial treatment with daily oral isoniazid (H), rifampicin (R), and pyrazinamide (Z), and ethambutol (E). After one month of hospitalization with a good response, she was discharged to continue treatment at home. However, 45 days later, she developed paresthesia and hypoesthesia of the lower limbs, along with hypogastrium and lumbar pain, which progressed to spastic paraparesis. She was hospitalized again and quickly referred to a tertiary hospital in Medellín. A thoraco-abdominal CT scan confirmed spinal cord involvement due to *Mycobacterium* spp.. Treatment was continued with daily HRZE for five months, however, due to her history of infections and lymphopenia, the regimen was extended to 41 months (22 months in daily HRZE followed by 19 months of HR twice a week), resulting in complete recovery. Soon after, prophylaxis was initiated with trimethoprim sulfamethoxazole (TMP-SMX) at the dose of 5 mg/kg/day (trimethoprim component), initially daily and later three times a week, and has remained with this prophylaxis until today. She was subsequently referred to the outpatient clinic of the Group of Primary Immunodeficiencies at the University of Antioquia in Medellin.

From the age of 30 to 40, she experienced three episodes of recurrent herpes zoster on her right thigh, successfully treated with oral and topical acyclovir. In recent years, she has had recurrent cold sores, oral thrush, and herpes, effectively managed with topical and oral antifungals and antivirals. At age 38, due to persistent heartburn, an upper gastrointestinal (GI) tract endoscopy diagnosed her with gastritis and chronic esophagitis, with a biopsy positive for *Helicobacter pylori*. She received clarithromycin, omeprazole, and amoxicillin, resulting in partial improvement. She was later diagnosed with non-erosive peptic esophagitis and chronic antral erythematous gastropathy, for which she has been receiving therapy with proton pump inhibitors and intermittent antacids.

At age 46, she presented with symptoms including lip inflammation, blisters, and oral ulcers affecting the tongue, cheeks, and gums, accompanied by friable muguet-type crusts. Additionally, she exhibited generalized erythematous and pruritic lesions on her skin. She was diagnosed with herpes simplex infection, oral candidiasis, and generalized erythema multiforme. Treatments included oral acyclovir (400mg four times daily for 10 days), topical 5% acyclovir, fluconazole (150mg daily for 10 days), and oral nystatin ($1x10^5$ U/mL; 5mL every 6 hours for 7 days), which led to the resolution of the lesions. She experienced further episodes of oral herpes, which responded well to acyclovir treatment.

At age 56, she presented with fever, nasal congestion, rhinorrhea, diaphoresis, anosmia, ageusia, asthenia, and adynamia. A SARS-CoV-2 infection was suspected and later confirmed by PCR. She received outpatient symptomatic treatment and fully recovered after 20 days

without requiring oxygen supplementation or hospitalization. Subsequently, she received two doses of the Pfizer BioNTech COVID-19 vaccine without adverse events. Neither her parents nor her siblings reported a history of immune-mediated diseases. (Supplemental Table 1).

Patient 3 (P3) is a 58-year-old female born in 1966 to consanguineous Japanese parents. She had a 40-year history of numerous verrucae on her limbs. She has had no remarkable medical history of other infectious diseases, experienced no side effects from vaccinations, including BCG vaccine. She developed appendicitis at age 22. Laboratory evaluation revealed mild lymphocytopenia (782 cells/mm3) with CD4 T-cell lymphopenia (45 cells/mm3). Serologic testing for anti–human immunodeficiency virus (anti-HIV) antibodies was negative. HPV typing with a degenerating polymerase chain reaction (PCR) method of three different sites (including verruca vulgaris– and verruca plana–like lesions) detected HPV-7 in all lesions. She has been treated with 10mg/day etretinate for five years, resulting in some improvement in her warts, with no observed complications from the medication. At age 53, she developed squamous cell carcinoma (SCC) on the left middle finger, which led to amputation, and SCC in situ on the buttock, which was surgically removed. In addition to generalized verrucosis and skin cancers, she developed a cerebral infarction, and tested positive for anti-phospholipid antibodies (anti-cardiolipin IgG and anti-beta2 GP1 IgG), leading to a diagnosis of anti-phospholipid syndrome. She has been treated with warfarin for anti-phospholipid syndrome for two years. Her parents, three siblings, and two children did not show similar phenotypes. Her father died of cholangiocarcinoma. **(Supplemental Table 1)**.

Patients 4, 5, and 6 (P4, P5, and P6): are siblings from a consanguineous Arabic family born and living in Israel. P4 is a 60-year-old male, P5 is a 55-year-old female, and P6 is a 48-year-old female, born in 1964, 1969, and 1976, respectively. They were described in 2015 by Horev and colleagues (1). All patients reported the onset of verrucous skin lesions beginning in their twenties. They all exhibited cutaneous manifestations, including deep palmoplantar warts and verrucous papules on the dorsal aspect of their fingers and toes. Two of them (P4 and P5) also presented flat warts (tinea versicolor-like lesions), one (P4) had seborrheic keratosis-like warts, and one (P4) developed nonmelanoma skin cancers. All patients tested positive for HPV-3 (samples taken from seborrheic keratosis-like, tinea versicolor-like, and verruca vulgaris-like lesions). Additionally, P4 and P5 presented with cryptococcal meningitis. Complete blood counts in all three patients revealed a marked decrease of $CD3^{+}CD4^{+}T$ lymphocytes and $CD3^{+}CD8^{+}T$ lymphocytes (1).

IL-7R deficient patient: This patient was a five-month-old boy born to unrelated Caucasian parents, who was taken to the emergency room (ER) for severe chickenpox with diffuse and infected lesions. The child was born at term with normal birth measurements. The parents reported fold mycosis since birth, but the patient otherwise had no particular medical history, and his height-weight growth chart was satisfactory. The infected chickenpox lesions were initially treated with local antibiotics, and the patient was discharged. His clinical state deteriorated, and he was brought back to the ER in respiratory distress and hemodynamic shock, with chickenpox lesions covering his entire body. A chest X ray showed a diffuse pulmonary infiltrate, and VZV tests on the skin, blood, respiratory secretions, and cerebrospinal fluid were

positive. A diagnosis of multisystemic varicella and SCID was strongly suspected. Blood immunophenotyping revealed an absence of T lymphocytes, normal B-cell levels, and low levels of NK cells, leading to the conclusion that the patient had $T^{-}B^{+}NK^{low}$ SCID. Blood immunoglobulin tests were negative (for IgG, IgA and IgM). Next-generation sequencing of genes known to be involved in immunodeficiencies confirmed the diagnosis by revealing a combination of a monoallelic variant in exon 3 leading to a stop codon (c.354C>A ; p.Cys118*) and a monoallelic deletion of exon 3 of the *IL7R* gene (NM_002185.4). The patient received antiviral therapy and underwent hematopoietic stem cell transplantation (HSCT) with his sister as a matched related donor. Unfortunately, the patient died from severe pulmonary immune reconstitution inflammatory syndrome in a context of high rates of VZV replication.

Supplemental Methods

Whole-exome sequencing (WES), variant filtering, and Sanger sequencing

Human genomic DNA (gDNA) was isolated from the pellets obtained from Ficoll-HiPaque Plus gradient centrifugation of PBMCs from whole blood or cell lines. The cells were lysed in extraction buffer and incubated overnight at 37°C. The DNA was isolated by phenol/chloroform extraction, precipitated in ethanol, and resuspended in 10 mM Tris, pH 7.4, 1 mM EDTA. WES was performed on 3 μg of whole-blood gDNA from patients (P1, P2, P3) and their relatives. The DNA was sheared with a Covaris S2 Ultrasonicator (Covaris). We used a SureSelect Human All Exome V4 (51M) or V5 (50M) capture kit and the HiSeq2000/2500 (Illumina) sequencing platform, according to the manufacturer's instructions. The reads were aligned with the human reference genome (hg19 and hg20 build), with BWA aligner were then recalibrated and annotated with the Genome analysis toolkit (GATK), PICARD (http://picard.sourceforge.net/) tools and ANNOVAR. The homozygosity rate was estimated from the patients' genomic DNA, as previously described (2). Minor allele frequencies (MAFs) in the general population, as reported in gnomAD database v2.1.1, and precomputed combined annotation–dependent depletion (CADD) scores (v1.3) were used for variant filtering. The mutation significance cutoff (MSC) was calculated as previously described (3). For the verification of variants, exons and flanking regions were amplified from DNA with the DreamTaq DNA polymerase. They were then sequenced by the Sanger method with the Big Dye Terminator v3.1 kit (Thermo Fisher Scientific), and subjected to capillary electrophoresis (#A30469, Applied Biosystems 3500xL system, Thermo Fisher Scientific).

Principal component analysis (PCA)

PCA was conducted on the WES data of 370 individuals from our in-house exome database and 746 unaffected individuals from the 1000 Genomes Project Phase 1 as a reference, as previously described (2). The analysis was restricted to variants covered by the Agilent SureSelect V1 system with a MAF greater than 5%. The principal components were obtained with PLINK software (4).

mRNA purification, RT-PCR and RT-qPCR

Total RNA was extracted from the indicated cells with the Plus Mini Kit (#74136, Qiagen) or the Quick-RNA MicroPrep Kit (#R1051, Zymo). The remaining genomic DNA was removed by extraction on a column or by DNase digestion. RNA was reverse-transcribed with oligodT

primers (#18418012 Thermo Fisher Scientific) and SuperScript II reverse transcriptase (#18064014 Thermo Fisher Scientific) or with the High-Capacity RNA-to-cDNA Kit (#4387406, Applied Biosystems), according to the manufacturer's protocol. qPCR was performed on cDNA with *Taq*Man Fast Universal PCR Master Mix (2X), no AmpErase UNG (#4352042, Thermo Fisher Scientific) on a 7500 Real-Time PCR System (Applied Biosystems) or *Taq*man ViiA7, with the following probes, all from Thermo Fisher Scientific: *IL7* exons 2-3 (Hs00174202_m1) and *GUSB* (#1702016). The data are displayed as the 2-DCt after normalization relative to GUS (endogenous control) expression (DCt).

Single-cell RNA sequencing on primary leukocytes

Cryopreserved PBMCs from P1-P3, and P6, one healthy local control, and one healthy Colombian control were analyzed by single-cell RNA sequencing (scRNASeq) as previously described (5). In addition to the data generated through this study, we integrated previously published data for healthy donors (*n*=8, one of whom was tested twice) in six batches of experiments. Briefly, thawed cells were washed with medium and filtered through a 70 µm-mesh MACS SmartStrainer (Miltenyi Biotec, Cat: 130-098-462) to remove large debris. Cells were then washed three times with PBS plus 0.5% FBS and filtered again through a 40 µm-mesh Falcon Cell Strainer (Corning, Cat: 352340) before use for single-cell capture on the 10X Genomics Chromium chip. Libraries were prepared with the Chromium Single-Cell 3' Reagent Kit (v3 Chemistry) and sequenced with an Illumina NovaSeq 6000 sequencer. Sequences were subjected to preprocessing with CellRanger. Approximately 10,000 cells were sequenced per sample. The data generated during this study were analyzed in an integrative manner with historical controls and publicly available control PBMC datasets downloaded from the 10X Genomics web portal (https://support.10xgenomics.com/single-cell-gene-expression/datasets). Data were first manually filtered on the basis of common quality-control metrics. Filtered data were then integrated with Harmony (6). Two sequential graph-based clusterings were performed. The first-round clustering identified general leukocyte subsets, whereas the second-round clustering identified memory and effector T-lymphocyte subsets and NK lymphocytes with a sufficiently high resolution. Clusters were identified on the basis of canonical marker gene expression with the aid of the SingleR pipeline (7) guided by the MonacoImmuneData (8). The CITE-Seq datasets obtained from 10X also provided information about the identity of each cluster. Public datasets from 10X were excluded from subsequent analyses. Pseudobulk principal component analysis (PCA) was conducted on the normalized read counts obtained through variance-stabilizing transformation (VST) with batch correction, with the *removeBatchEffect* function implemented in limma (9). Pseudobulk differential expression analysis was conducted with DESeq2 (10). Geneset enrichment analysis (GSEA) was conducted with the fgsea package, by projecting the fold-change ranking onto various MSigDB genesets (http://www.gsea-msigdb.org/gsea/msigdb/genesets.jsp). All analyses were performed in R v4 (http://www.R-project.org/).

RNA sequencing analysis on T-cell blasts

We sequenced mRNA on an Illumina Nextseq system with a read length of 75 bp and a read depth of 20~30 million reads. All FASTQ files passed quality control and were aligned with the GRCh38 reference genome with STAR (2.6.1d). BAM files were converted to a raw-count

expression matrix with featurecount. Raw count data were normalized with DEseq2. The ensemble IDs targeting multiple genes were collapsed (average), and a final data matrix gene was generated for downstream analysis.

High-throughput sequencing (HTS) of *TRA* and *TRB*

DNA was extracted from whole blood samples from the IL-7 deficient patients (P1 and P2). The TCRa (*TRA*) and TCRb (*TRB*) rearranged genomic products were amplified by multiplex polymerase chain reaction (PCR), with DNA as the template (Adaptive Biotechnologies Seattle, WA). The Adaptive Biotechnologies system uses assay-based and computational techniques to minimize PCR amplification bias. The assay is quantitative, and the frequency of a given *TRA*/*TRB* sequence is representative of the frequency of that clonotype in the original sample. The PCR products were sequenced on the Illumina HiSeq platform. Custom algorithms were used to filter the raw sequences for errors and to align the sequences with reference genome sequences. The data were then analyzed with the ImmunoSeq set of online tools. The frequency of productive and nonproductive *TRA* or *TRB* rearrangements was analyzed within both unique and total *TRA* or *TRB* templates obtained from T lymphocytes. The distribution and frequency of individual clonotypes (including *TRAV* to *TRAJ* and *TRBV* to *TRBJ* pairings) and the use of individual genes were analyzed within unique sequences. Heat-map representations of the frequencies of individual *TRAV*/*TRBV* to *TRAJ*/*TRBJ* genes and sequence overlaps were produced with R software version 3.6.3 (2020-02-29). The ImmunoSeqTM set of online tools was used to analyze the Gini-Simpson index of Diversity and Simpson clonality indices. Treemaps were generated with R software version 3.6.3. Each dot represents a CDR3 and the size of the dot represents the frequency of that specific CDR3 within the sample.

TREC quantification

TREC were quantified by nested multiplex qPCR, with an adapted version of a published protocol (11). Briefly, 3 x 10^6 PBMCs were resuspended in lysis buffer (20 mM Tris-HCl pH 8.0 (Sigma Aldrich); 0.1% NP40; 0.1% Tween 20 (Sigma Aldrich); 200 µg/mL proteinase K (Eurobio)) and incubated for 30 min at 56°C. Proteinase K was inactivated by heating for 15 min at 95°C. Multiplex polymerase chain reaction (PCR) amplification was performed for sjTREC together with the CD3γ chain, in a final volume of 100 µL (initial denaturation for 10 minutes at 95°C, then 22 cycles of 30 seconds at 95°C, 30 seconds at 60°C, 2 minutes at 72°C) with the outer 3'/5' primer pairs described in Supplemental Table 5. The qPCR conditions in the LightCycler experiments, using the inner primer pairs described in Supplemental Table 5 and performed on 1/100th of the initial PCR products, were: initial denaturation for one minute at 95°C, followed by 40 cycles of 1 second at 95°C, 10 seconds at 60°C, and 15 seconds at 72°C. Fluorescent signals were quantified at the end of the annealing steps. The sjTREC and CD3γ LightCycler quantifications were performed in independent experiments, with the same first-round serial dilution standard curve obtained with plasmids containing the CD3γ amplicon, cloned together with the sjTREC, amplicons (12).

Cell culture

Peripheral blood mononuclear cells (PBMCs) were isolated by Ficoll-Hypaque density gradient centrifugation (Amersham-Pharmacia-Biotech). EBV-B cells were cultured in RPMI-1640

medium supplemented with 10% fetal calf serum (FCS). For T-blast induction, PBMCs were cultured in ImmunoCult™-XF T-Cell Expansion Medium (Stemcell) in the presence of ImmunoCultTM Human CD3/CD28/CD2 T-cell activator (12.5 µL/mL) and human recombinant IL-2 (100 ng/mL, Novartis).

Site-directed mutagenesis, transient and stable transfection

Empty vector (EV) and a plasmid containing the DDK-tagged *IL7* cDNA were obtained commercially (#RC208274, Origene). Constructs carrying single-nucleotide mutant alleles were generated from this plasmid by mutagenesis with appropriate primers, with the Pfu Ultra II Fusion HS DNA (#600674, Agilent) polymerase, followed by digestion with *Dpn*I (#R0176L, New England Biolab). For evaluations of the reinitiation of translation, methionine codons were mutated into alanines (ATG>GCG). Plasmids were amplified in competent cells of *E. coli* (#C3019H, New England Biolab) and purified with a maxiprep kit (#12663, Qiagen). HEK293T cells were transiently transfected with the various constructs at a concentration of 2.5 µg/mL, with the Lipofectamine LTX kit (#15338100, Thermo Fisher Scientific) in accordance with the manufacturer's instructions.

Supernatant-induced STAT5 phosphorylation in TAIL7 and BaF3 cells

We cultured TAIL7 cells at a density of 2 x 10^6 viable cells/mL in RPMI (GibcoBRL, Invitrogen) supplemented with 10% fetal bovine serum (FBS) (GibcoBRL, Invitrogen) and 20 ng/mL IL-7. BaF3 cells stably transduced with human IL-7R, and TAIL7 cells were starved overnight in medium without FBS. The cells were then counted and plated at a density of 5 x 10^5 cells/well in 96-well V-bottom plates (Thermo Fisher Scientific), in 100 µL RPMI (GibcoBRL, Invitrogen); cells were either left unstimulated or stimulated with 50 µL of 1:10 dilutions of supernatants from human embryonic kidney (HEK) 293T cells transiently transfected with pCMV6 carboxy-terminal DDK tag expression vectors containing the WT or the mutant (c.3G>A, c.205A>T, c.284del, c.8A>G, c.14C>A, and c.52G>A) *IL7* alleles or with an empty pCMV6 vector (EV), or incubated with supernatants from resting EBV-B cells from P2 or healthy controls. The cells were incubated with 20 ng/mL rhIL-7 (Miltenyi Biotec) for 15 minutes at 37°C and were then fixed and permeabilized with a fixation/permeabilization kit (eBioscience). Cell viability was assessed with the LIVE/DEAD™ Aqua Fixable Dead Cell Stain Kit (Thermo Fisher Scientific) and STAT5 phosphorylation (p-STAT5 levels) was assessed by intracellular staining with Phospho-Flow PE Mouse Anti-p-STAT5 (pY694) antibody (BD-Biosciences). Data were collected with a Gallios flow cytometer (Beckman-Coulter) and analyzed with FlowJo software v.10.6.2 (Becton-Dickinson).

***In vitro* differentiation of effector memory $CD4^+$ T lymphocytes**

Memory $CD4^+$ T lymphocytes (defined as $CD45RA^-CCR7^+CD4^+$) were isolated (>98% purity) from healthy controls or the patients with a FACS Aria Flow cytometer. Cells were cultured with T-cell activation and expansion beads (anti-CD2/CD3/CD28; Miltenyi Biotec) in polarizing conditions. After five days of culture, cytokine production was assessed by ELISA or LEGENDplex Th Cytokine Panel (741027) on the culture supernatant. Viability was assessed by FACS after Zombie dye fixation (Biolegend).

In vitro T-cell proliferation assay

Fresh PBMCs from P1, P2, and healthy controls matched for ethnicity, age and sex were labeled with CFSE (Invitrogen) and plated in 96-well plates (2.5 x 10^6/mL) in 200 µL RMPI supplemented with 10% FCS. T-cell proliferation was assessed after four days of stimulation with T-cell activation and expansion beads (anti-CD3/CD28 antibody-coated; Miltenyi Biotec), PHA (Sigma, #L2646, 1 µg/mL), and 10 ng/mL rIL-2 (Thermo Fisher Scientific). Cells were collected and labeled with mAbs against CD3 (RPA-T4, BD) and stained with LIVE/DEAD® Fixable Aqua stain.

Cell lysis and immunoblotting

Total protein extracts were prepared by mixing cells with lysis buffer (50 mM Tris pH 7.4, 150 mM NaCl, 2 mM EDTA, 0.5% Triton X-100) and incubating for 30 minutes at 4°C. The cells were centrifuged for 10 minutes at 16000 x *g*, and the supernatant was collected for immunoblotting. A mixture of protease and phosphatase inhibitors was added to the buffers immediately before use: aprotinin (Sigma, 10 mg/mL), PMSF (Sigma, 1 mM), leupeptin (Sigma, 10 mg/mL), phosSTOP (Sigma, 1x) di-isopropylfluorophosphate (DFP, Sigma, 5 mM). The proteins were separated by SDS-PAGE and immunoblotting was performed with Abs against the GAPDH-tag (FL335, Santa Cruz) and IL-7 (ab193358, Abcam).

Deep immunophenotyping

Freshly thawed PBMCs (1.0~1.5 × 10^6 cells) from P1, P2, P3, and P6 were immunophenotyped by flow cytometry, as previously described (13). Historical data for healthy controls were compiled. Briefly, freshly thawed PBMCs (1.2 × 10^6 cells) were simultaneously stained with LIVE/DEAD Fixable Blue dye (Cat: L23105, 1:800; Thermo Fisher Scientific in PBS) and blocked by incubation with FcR blocking reagent (Miltenyi Biotec, 1:25) on ice for 15 min. After washing, cells were surface-stained with the following reagents on ice for 30 min: Brilliant Stain Buffer Plus (Cat: 566385; BD Biosciences, 1:5), anti-γδTCR-BUV661 (Cat: 750019; BD Biosciences, Clone: 11F2, 1:50), anti-CXCR3-BV750 (Cat: 746895, Clone: 1C6, 1:20; BD Biosciences), and anti-CCR4-BUV615 (Cat: 613000, Clone: 1G1, 1:20; BD Biosciences) antibodies. Cells were then washed and surface-stained with the following reagents on ice for 30 min: 5-OP-RU-loaded MR1 tetramer-BV421 (NIH Tetramer Core Facility, 1:100), anti-CD141-BB515 (Cat: 565084, Clone: 1A4, 1:40; BD Biosciences), anti-CD57-FITC (Cat: 347393, Clone: HNK-1, 3:250; BD Biosciences), anti-Vδ2-PerCP (Cat: 331410, Clone: B6, 3:500; BioLegend), anti-Vα7.2-PerCP-Cy5.5 (Cat: 351710, Clone: 3C10, 1:40; BioLegend), anti-Vδ1-PerCP-Vio700 (Cat: 130-120-441, Clone: REA173, 1:100; Miltenyi Biotec), anti-CD14-Spark Blue 550 (Cat: 367148, Clone: 63D3, 1:40; BioLegend), anti-CD1c-Alexa Fluor 647 (Cat: 331510, Clone: L161, 1:50; BioLegend), anti-CD66b-APC (Cat: 305118, Clone: G10F5, 1:50; BioLegend), anti-CD38-APC-Fire 810 (Cat: 356644, Clone: HB-7, 3:100; BioLegend), anti-CD27-APC H7 (Cat: 560222, Clone: M-T271, 1:50; BD Biosciences), anti-CD127-APC-R700 (Cat: 565185, Clone: HIL-7R-M21, 1:50; BD Biosciences), anti-CD19 Spark NIR 685 (Cat: 302270, Clone: HIB19, 3:250; BioLegend), anti-CD45RA-BUV395 (Cat: 740315, Clone: 5H9, 3:250; BD Biosciences), anti-CD16-BUV496 (Cat: 612944, Clone: 3G8, 3:500; BD Biosciences), anti-CD11b-BUV563 (Cat: 741357, Clone: ICRF44, 1:100; BD Biosciences), anti-CD56-BUV737 (Cat: 612767, Clone: NCAM16.2,

3:250; BD Biosciences), anti-CD4-cFluor 568 (Cytek, Clone: SK3, 3:250), anti-CD8-BUV805 (Cat: 612889, Clone: SK1, 3:250; BD Biosciences), anti-CD11c-BV480 (Cat: 566135, Clone: B-ly6, 1:40; BD Biosciences), anti-CD45-BV510 (Cat: 563204, Clone: HI30, 3:250; BD Biosciences), anti-CD33-BV570 (Cat: 303417, Clone: WM53, 3:250; BioLegend), anti-iNKT-BV605 (Cat: 743999, Clone: 6B11, 1:25; BD Biosciences), anti-CD161-BV650 (Cat: 563864, Clone: DX12, 1:25; BD Biosciences), anti-CCR6-BV711 (Cat: 353436, Clone: G034E3, 3:250; BioLegend), anti-CCR7-BV785 (Cat: 353230, Clone: G043H7, 1:40; BioLegend), anti-CD3-Pacific Blue (Cat: 344824, Clone: SK7, 3:250; BioLegend), anti-CD20-Pacific Orange (Cat: MHCD2030, Clone: HI47, 1:50; Invitrogen), anti-CD123-Super Bright 436 (Cat: 62-1,239-42, Clone: 6H6, 1:40; Invitrogen), anti-Vβ11-PE (Cat: 130-123-561, Clone: REA559, 3:500; Miltenyi Biotec), anti-CD24-PE-Alexa Fluor 610 (Cat: MHCD2422, Clone: SN3, 1:25; Invitrogen), anti-CD25-PE-Alexa Fluor 700 (Cat: MHCD2524, Clone: 3G10, 1:25; Invitrogen), anti-CRTH2-Biotin (Cat: 13-2949-82, Clone: BM16, 1:50; Invitrogen), anti-CD209-PE-Cy7 (Cat: 330114, Clone: 9E9A8, 1:25; BioLegend), anti-CD117-PE-Dazzle 594 (Cat: 313226, Clone: 104D2, 3:250; BioLegend), and anti-HLA-DR-PE-Fire 810 (Clone: L243, 1:50; BioLegend) antibodies. After washing, cells were further incubated with streptavidin-PE-Cy5 (Cat: 405205, 1:3,000; BioLegend) on ice for 30 min. Cells were then washed, fixed with 1% paraformaldehyde/PBS, washed again, and acquired with an Aurora cytometer (Cytek). Subsets were manually gated with FlowJo v10 (FlowJo, LLC) and further analyzed in R. The cellular composition was visualized with the data downsampled to 50,000 cells per sample through Uniform Manifold Approximation and Projection (UMAP) based on the expression levels of the following markers: CD3, CD4, CD8, CD11c, CD14, CD16, CD19, CD20, CD56, CD117, CD123, CD127, CD161, CRTH2, HLA-DR, γδTCR, Vδ1, Vδ2, iNKT, and MR1.

Flow cytometry for NK cell and ILC subsets

Flow cytometry analysis was performed on PBMCs labeled with the following antibodies: biotinylated anti-human antibodies against CD1a (REA736; Miltenyi Biotec), CD14 (REA599; Miltenyi Biotec), CD66abce (REA1230; Miltenyi Biotec), CD123 (REA918; Miltenyi Biotec), CD203c (REA826; Miltenyi Biotec), CD303 (REA693; Miltenyi Biotec) and FceRIa (AER-37(CRA-1); BioLegend), together with streptavidin BV570 or PE-Cy5 (BioLegend); and conjugated anti-human CD45 Alexa Fluor 532 (HI30; Thermo Fisher Scientific, CD34 Red 718 (581, BD Biosciences), CD3 BB790-P (UCHT1; BD Biosciences), CD8b BV480 (2ST8.5H7; BD Biosciences), CD4 NovaFluor Blue 660-120S (SK3; Thermo Fisher Scientific), CD5 PerCP (UCHT2; BioLegend) or BUV615 (UCHT2; BD Biosciences) CD19 BV570 or PE-Cy5 (HIB19; BioLegend), CD7 BUV496 or BV510 (M-T701; BD Biosciences), CD94 BUV661 or BV750 (HP-3D9; BD Biosciences), CD16 PE-Alexa Fluor 700 (3G8; Thermo Fisher Scientific), CD56 BB755-P or BUV661 (NCAM16.2; BD Biosciences), NKG2A APC-Fire 750 (S19004C; BioLegend), NKG2C PE-Vio615 (REA205; Miletnyi Biotec), CD57 BV785 (QA17A04; BioLegend), CD127 PE-Cy7 (eBioRDR5; Thermo Fisher Scientific), CD161 BUV395 (HP-3G10; BD Biosciences), CD25 BV750 or BUV563 (2A3; BD Biosciences), CD117 BV605 (104D2; BioLegend), CRTH2 Alexa Fluor 647 (BM16; BD Biosciences), CD45RA BUV496 (HI100; BD Biosciences), NKp44 BB630-P2 (p44-8; BD Biosciences) and EOMES (WD1928; Thermo Fisher Scientific) Abs. Fc receptors were blocked with IgG from human serum (Millipore Sigma) and non-specific dye binding was inhibited with CellBlox

blocking buffer (Thermo Fisher Scientific). Surface membrane staining was performed in Brilliant Stain Buffer (BD Biosciences). EOMES was stained with the Foxp3 staining buffer set (Thermo Fisher Scientific) according to the manufacturer's instructions. LIVE/DEAD Blue fixable viability dye (Thermo Fisher Scientific) was used to exclude dead cells. Samples were fixed in 2% PFA, resuspended in 2% FCS and 5 mM EDTA in PBS, acquired on an ID7000 (Sony Biotechnology) machine with ID7000 Software v1.2.0.28121 (Sony Biotechnology) and analyzed with FlowJo 10.8.1 (BD Biosciences).

Phage immunoprecipitation sequencing (PhIP-Seq)

The VirScan phage library used for PhIP-Seq in this study has been described elsewhere (14). Briefly, a library of oligonucleotides encoding 56-amino acid peptides tiling across the genomes of 206 viral species was synthesized on a releasable DNA microarray and inserted into the T7 phage for expression. Patient serum was added to the phage library and immunoprecipitation was performed with Protein A and G beads. Enriched peptides were identified by PCR amplification and sequencing of the peptide cassette consisting of the cloned oligonucleotide embedded in immunoprecipitated T7 phages on the Illumina platform.

Protein microarray

Protein microarrays (HuProtTM, CDI laboratories) were used according to an established protocol (15) starting with incubation in 5 mL blocking buffer (2% bovine serum albumin with 0.05% Tween 20 in phosphate-buffered saline, PBS) for 90 min. They were then incubated overnight in 5 mL blocking buffer per microarray with serum from a patient or blood donor diluted 1:2000. Microarrays were then washed five times for five minutes each, with 5 mL PBS-T (PBS + 0.05 % Tween 20). Alexa Fluor 647 goat anti-human IgG (Thermo Fisher Scientific Cat#A-21445, RRID:AB_2535862) and Dylight® 550 goat anti-GST (Columbia Biosciences Corporation Cat#D9-1310) were added to blocking buffer (1:2000 and 1:10 000 respectively) and each microarray was incubated in 5 mL of this mixture for 90 minutes. Washing was then repeated, as previously described. Incubations and washes were performed on an orbital shaker, with aluminum foil to protect against light during the steps following the addition of secondary antibodies. Finally, the microarrays were immersed in deionized water, dried by centrifugation for about 30 seconds, and this washing and drying procedure was then repeated twice. The arrays were scanned the same day with an Innoscan 1100AL Fluorescence scanner (Innopsys) using Mapix 9.1.0, and the resulting images were analyzed with the Jan 18- 22 Huprot v4.0 Genepix Array List file and either GenePix Pro 5.1.0.19 or GenePix Pro 7. Normalization was applied to compensate for signal variation between experiments. Data from healthy donors obtained in separate protein array experiments were included. Signal intensities were extracted by subtracting the local background. Autoantigens identified in patients with APS-1 were extracted as previously described (15-18). Protein array analyses were performed on plasma from two patients (P1 and P2) with IL-7 deficiency studied here. We also performed similar analyses on plasma from healthy donors (n=12) matched with the two patients for sex and age.

ELISA

Supernatants from HEK293T cells and SV40-transformed fibroblasts, and plasma samples were assessed for the determination of IL-7 levels by Quantikine (#HS750, R&D Systems) or Duoset

ELISA (#DY207, R&D Systems) in accordance with the manufacturer's protocol. Supernatant from T-blast were assessed for determination of IFN-γ, TNF, IL-2, IL-4, IL-9, IL-10, IL-13, IL-17A, and IL-17F by LEGENDplex Th Cytokine Panel (741027).

Thymus CT scan

We performed thymus assessment on the patients for whom a chest CT-scan was available (P1, P2, and P3). These scans were performed without contrast injection, and the thymic margins were assessed by multiplanar reconstruction. The thymus was measured in three planes: thickness and width in the axial plane through the aortic arch, greatest height in a coronal or sagittal oblique plane. We also included a healthy individual control matched with P1 for age and sex. This control was selected from scans performed at our center for polytrauma, excluding severe head trauma with coma or neurologic disorders and thoracic trauma (so as not to alter mediastinal anatomic reports).

Supplemental Acknowledgments: Generalized Verrucosis Japanese Consortium

Hideyuki Kosumi[33], Shota Takashima[33], Toshinari Miyauchi[33], Yi-Ting Huang[36], Toshifumi Nomura[33, 37], Teruki Yanagi[33, 38], Hsin-Yu Huang[39], Frank Po-Chao Chiu[40], Peng-Chieh Chen[41], Chao-Kai Hsu[39, 40, 41], Hideyuki Ujiie[33], and Hiroshi Shimizu[33]

[33]Department of Dermatology, Faculty of Medicine and Graduate School of Medicine, Hokkaido University, Sapporo, Japan

[36]School of Medicine, College of Medicine, National Cheng Kung University, Tainan, Taiwan

[37]Department of Dermatology, Institute of Medicine, University of Tsukuba, Tsukuba, Japan

[38]Department of Dermatology, University of the Ryukyus Graduate School of Medicine, Okinawa, Japan

[39]Department of Dermatology, National Cheng Kung University Hospital, College of Medicine, National Cheng Kung University, Tainan, Taiwan

[40]International Center for Wound Repair and Regeneration, National Cheng Kung University, Tainan, Taiwan

[41]Institute of Clinical Medicine, College of Medicine, National Cheng Kung University, Tainan, Taiwan

Supplemental References

1. Horev L, Unger S, Molho-Pessach V, Meir T, Maly A, Stepensky P, et al. Generalized verrucosis and HPV-3 susceptibility associated with CD4 T-cell lymphopenia caused by inherited human interleukin-7 deficiency. *J Am Acad Dermatol.* 2015;72(6):1082-4.
2. Belkadi A, Pedergnana V, Cobat A, Itan Y, Vincent QB, Abhyankar A, et al. Whole-exome sequencing to analyze population structure, parental inbreeding, and familial linkage. *Proc Natl Acad Sci U S A.* 2016;113(24):6713-8.
3. Itan Y, Shang L, Boisson B, Ciancanelli MJ, Markle JG, Martinez-Barricarte R, et al. The mutation significance cutoff: gene-level thresholds for variant predictions. *Nat Methods.* 2016;13(2):109-10.
4. Purcell S, Neale B, Todd-Brown K, Thomas L, Ferreira MA, Bender D, et al. PLINK: a tool set for whole-genome association and population-based linkage analyses. *Am J Hum Genet.* 2007;81(3):559-75.

5. Ogishi M, Arias AA, Yang R, Han JE, Zhang P, Rinchai D, et al. Impaired IL-23-dependent induction of IFN-gamma underlies mycobacterial disease in patients with inherited TYK2 deficiency. *J Exp Med.* 2022;219(10).
6. Korsunsky I, Millard N, Fan J, Slowikowski K, Zhang F, Wei K, et al. Fast, sensitive and accurate integration of single-cell data with Harmony. *Nat Methods.* 2019;16(12):1289-96.
7. Aran D, Looney AP, Liu L, Wu E, Fong V, Hsu A, et al. Reference-based analysis of lung single-cell sequencing reveals a transitional profibrotic macrophage. *Nat Immunol.* 2019;20(2):163-72.
8. Monaco G, Lee B, Xu W, Mustafah S, Hwang YY, Carre C, et al. RNA-Seq Signatures Normalized by mRNA Abundance Allow Absolute Deconvolution of Human Immune Cell Types. *Cell Rep.* 2019;26(6):1627-40 e7.
9. Ritchie ME, Phipson B, Wu D, Hu Y, Law CW, Shi W, et al. limma powers differential expression analyses for RNA-sequencing and microarray studies. *Nucleic Acids Res.* 2015;43(7):e47.
10. Love MI, Huber W, and Anders S. Moderated estimation of fold change and dispersion for RNA-seq data with DESeq2. *Genome Biol.* 2014;15(12):550.
11. Dion ML, Sekaly RP, and Cheynier R. Estimating thymic function through quantification of T-cell receptor excision circles. *Methods Mol Biol.* 2007;380:197-213.
12. Dion ML, Poulin JF, Bordi R, Sylvestre M, Corsini R, Kettaf N, et al. HIV infection rapidly induces and maintains a substantial suppression of thymocyte proliferation. *Immunity.* 2004;21(6):757-68.
13. Ogishi M, Yang R, Rodriguez R, Golec DP, Martin E, Philippot Q, et al. Inherited human ITK deficiency impairs IFN-gamma immunity and underlies tuberculosis. *J Exp Med.* 2023;220(1).
14. Xu GJ, Kula T, Xu Q, Li MZ, Vernon SD, Ndung'u T, et al. Viral immunology. Comprehensive serological profiling of human populations using a synthetic human virome. *Science.* 2015;348(6239):aaa0698.
15. Le Voyer T, Parent AV, Liu X, Cederholm A, Gervais A, Rosain J, et al. Autoantibodies against type I IFNs in humans with alternative NF-kappaB pathway deficiency. *Nature.* 2023;623(7988):803-13.
16. Philippot Q, Fekkar A, Gervais A, Le Voyer T, Boers LS, Conil C, et al. Autoantibodies Neutralizing Type I IFNs in the Bronchoalveolar Lavage of at Least 10% of Patients During Life-Threatening COVID-19 Pneumonia. *J Clin Immunol.* 2023;43(6):1093-103.
17. Goncalves D, Mezidi M, Bastard P, Perret M, Saker K, Fabien N, et al. Antibodies against type I interferon: detection and association with severe clinical outcome in COVID-19 patients. *Clin Transl Immunology.* 2021;10(8):e1327.
18. Frasca F, Scordio M, Santinelli L, Gabriele L, Gandini O, Criniti A, et al. Anti-IFN-alpha/-omega neutralizing antibodies from COVID-19 patients correlate with downregulation of IFN response and laboratory biomarkers of disease severity. *Eur J Immunol.* 2022;52(7):1120-8.

Supplemental Figure 1

A

WES analysis strategy	P1	P2	P3
Homozygosity rate	5.8%	1.0%	1.7%
Total number of variants	115,545	111,380	89,901
Number of variants MAF < 0.01(gnomAD 2.1)	7,004	6,880	5,670
Homozygous coding and essential splicing variants CADD > MSC; GDI < 12.41 Remove «Blacklist» HGID genes excluded DP <=2	41	17	25
Variants in common genes	*IL7* c.284del; (p.N95Ifs*11)		*IL7* c.3G>A

B

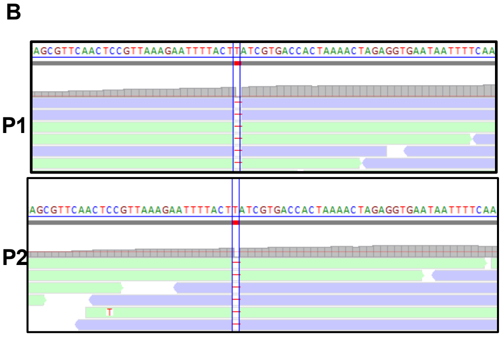

C

Approach	Estimated length	Number of genotypes per locus	Location on chromosome 8
GWAS InfiniumOmni2-5	21.67 Mb	15,930	From 8:59,025,114 to 8:80,695,081
WES	21 Mb	163	From 8:59,347,184 to 8:80,678,865
WGS	22.8 Mb	15,930	From 59,060 to 80,830,921
(last common ancestor) by *ESTIAGE* software	300 years ago (12 generations ago)	95% CI 175-900 years (7 generations - 36 generations)	

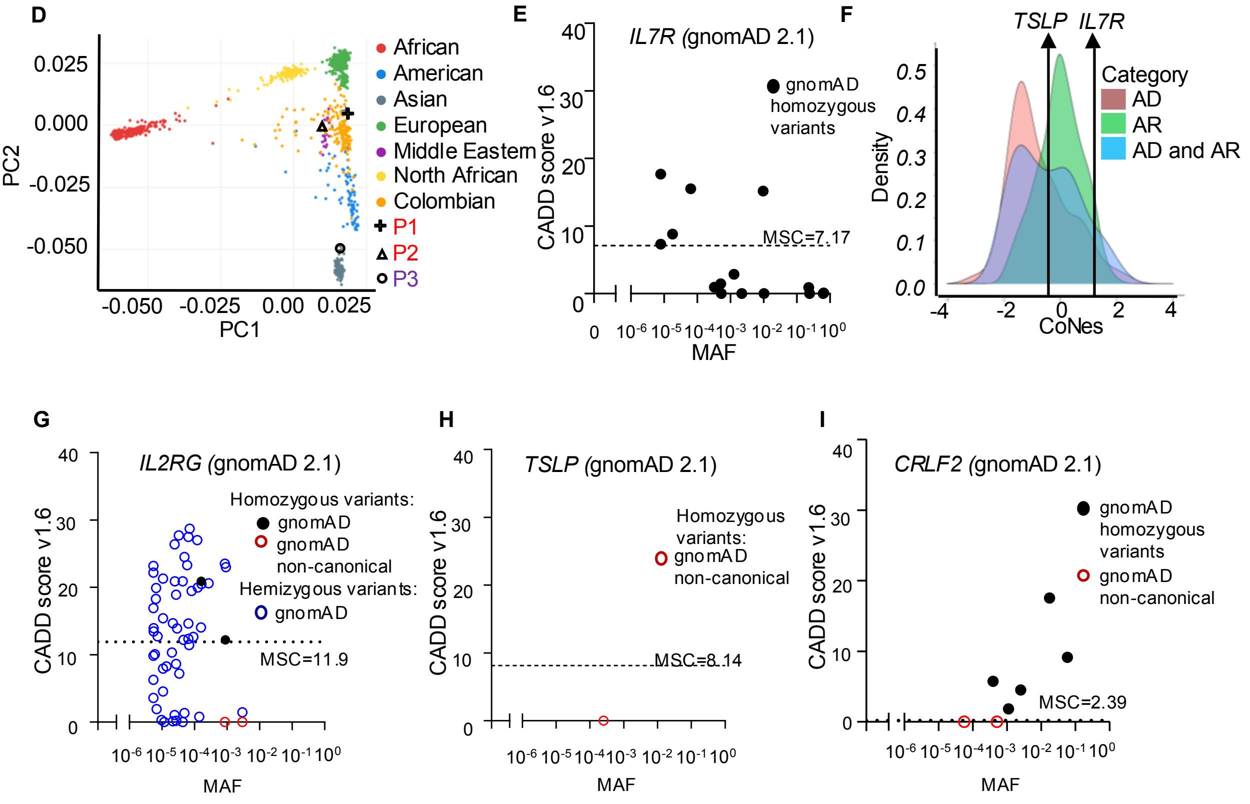

Supplemental Figures Legends

Supplemental Figure 1. Related to Figure 1

Private biallelic IL7 variants in six patients from four unrelated kindred

(A) WES strategy analysis for P1, P2, and P3. (B) Alamut viewer presentation of the region encompassing the c.284del mutant IL7 allele present in P1 and P2. (C) Haplotype common to P1 and P2 on WES, WGS and GWAS. (D) Principal component analysis of WES data from P1, P2, and P3 and our in-house WES database. (E) Combined annotation depletion-dependent (CADD) score vs. minor allele frequency (MAF) plot for all nonsynonymous IL7R variants present in the homozygous state in the gnomAD database (v2.1). The 99% mutation significance cutoff (MSC) is displayed (dotted line). (F) Consensus negative selection (CoNeS) of IL7R and TSLP. (G-I) CADD score vs. MAF plot for all nonsynonymous variants present in the homozygous and hemizygous states in the gnomAD database (v2.1) for IL2RG (G), TSLP (H), and CRLF2 (I). The 99% mutation significance cutoff (MSC) is displayed (dotted line).

Supplemental Figure 2

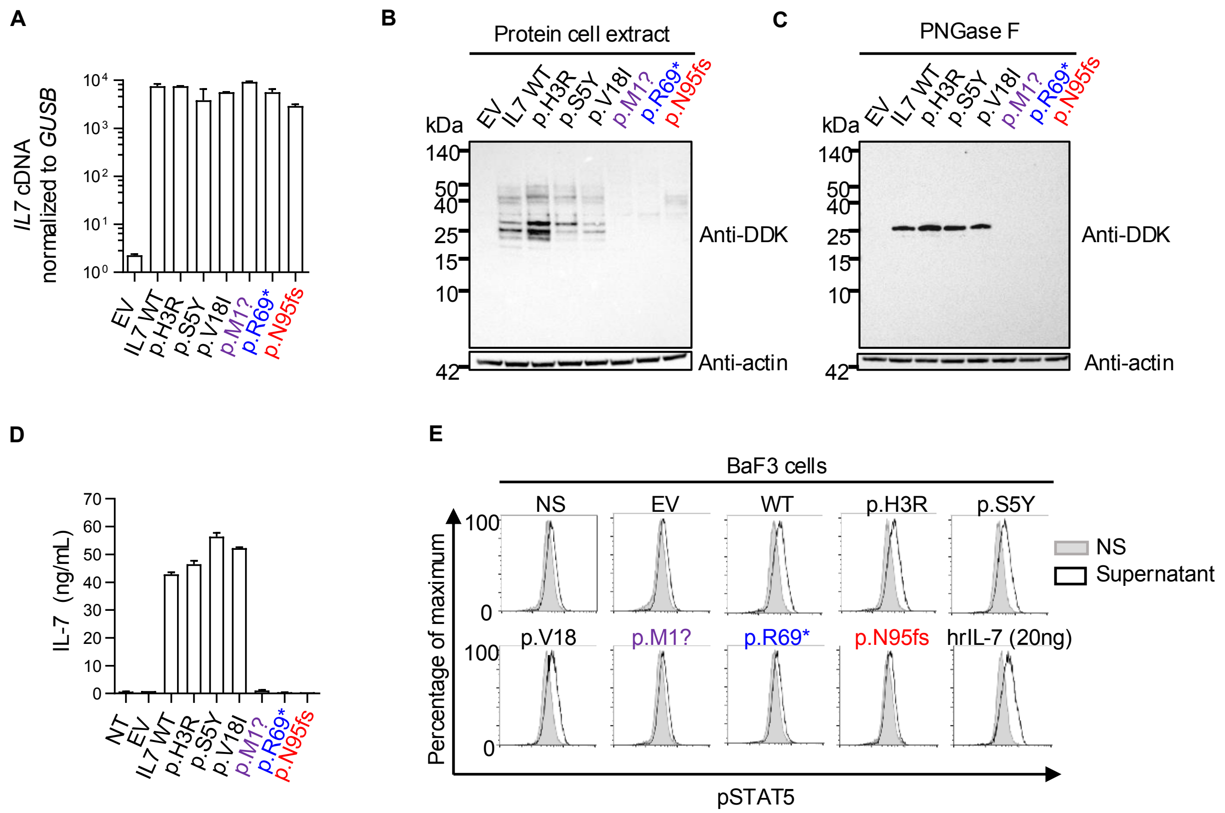

Supplemental Figure 2. Related to Figure 2

The IL7 mutant alleles are biochemically deleterious in an overexpression system.

(A) RT-qPCR on RNA extracted from HEK293T cells transfected with an empty vector (EV) or WT or mutant IL7 cDNAs. GUSB was used for normalization. (B-C) Western blot with an anti-DDK antibody on (B) total lysate, and (C) total lysate after PNGase F treatment. (D) IL-7 levels in the supernatants of HEK293T cells not transfected or transfected with WT or mutant IL7 cDNAs, or with empty vector (EV), as determined by ELISA. (E) STAT5 phosphorylation (pSTAT5) in BaF3 cells after 15 min of incubation with the supernatants of HEK293T cells transfected with C-terminally DDK-tagged IL7 WT, patients', or gnomAD IL7 mutant cDNAs or with an empty vector (EV). NS: non-stimulated (gray), supernatant (black line). Representative data from two independent experiments are shown.

Supplemental Figure 3

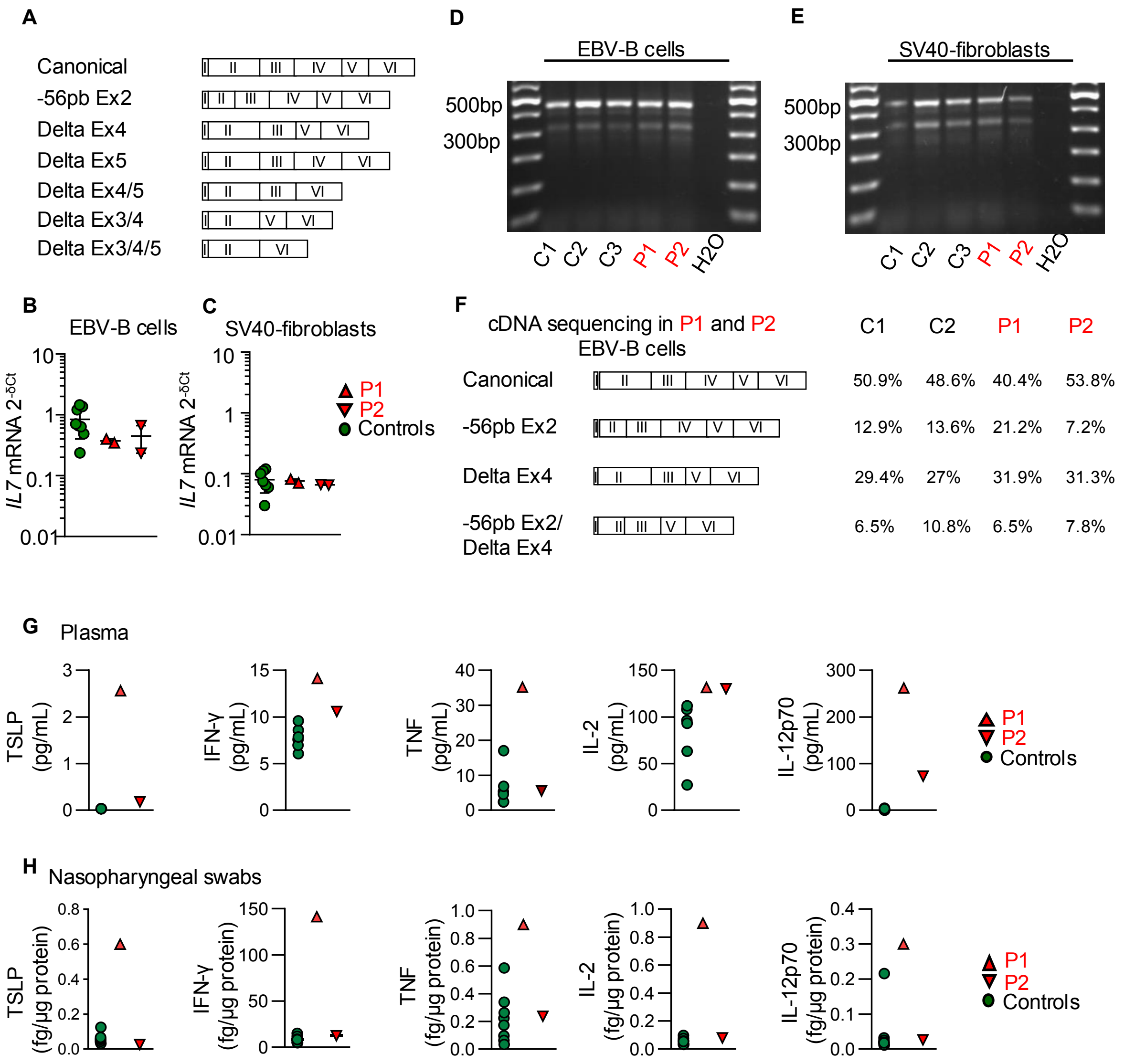

Supplemental Figure 3. Related to Figure 3

Lack of endogenous IL-7 detection.

(A) Schematic representation of seven previously described IL7 transcripts. (B-C) RT-qPCR for IL7 normalized against GUSB and the mean of controls (CTLs) on cDNA from (B) EBV-B cells (n=4 controls, P1 and P2), and (C) SV40-fibroblasts (n=3 controls, P1 and P2) from controls and patients; the bars indicate the mean. (D-E) Full-length IL7 cDNA amplification from (D) EBV-B cells and (E) SV40-fibroblasts from three controls (C1-C3), P1 and P2. H2O was used as a negative control. (F) Schematic representation of IL7 transcripts and their relative frequencies after cDNA cloning and sequencing in EBV-B cells from two healthy controls (C1 and C2), P1, P2. (G-H) TSLP, IFN-γ, TNF, IL-2, and IL-12p70 levels, as determined by ELISA, in plasma (G), and nasopharyngeal swabs (H) from healthy donors (n=5, green circles), and patients (red triangle for P1 and inverted red triangle for P2).

Supplemental Figure 4

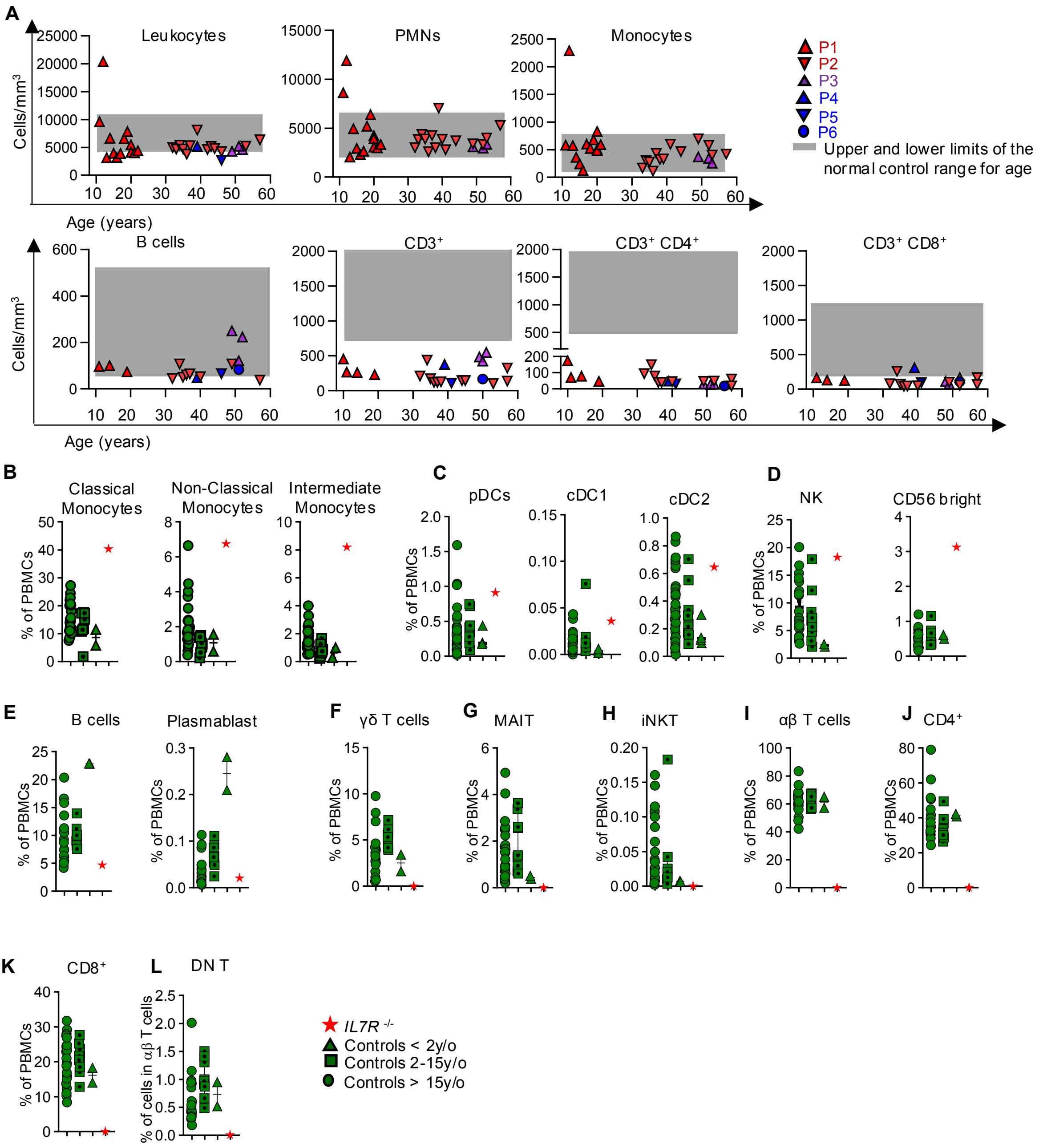

Supplemental Figure 4. Related to Figure 4

Impaired development of specific T-lymphocyte subsets.

(A) Follow-up of the counts for total leukocytes, polymorphonuclear neutrophils (PMNs), monocytes, B cells, CD3+, CD3+/CD4+, and CD3+/CD8+ cells in whole blood for the six IL-7 deficient patients (red triangle for P1, inverted red triangle for P2, purple triangle for P3, blue triangle for P4, inverted blue triangle for P5, blue circle for P6); the upper and lower limits of the normal range for age are shown in gray. (B-L) Frequencies of leukocyte subsets for a four-month-old IL-7R-deficient SCID patient (red star), healthy controls under the age of two years (green triangles), healthy controls aged two to 15 years (green square), and healthy controls over the age of 15 years (green dots), as assessed by CyTOF. Frequency of (B) classical monocytes (CD14+ CD16−), non-classical monocytes (CD14dim CD16+), and intermediate monocytes (CD14+ CD16+), (C) plasmacytoid dendritic cells (pDCs) (Lin-HLA-DR+CD11c-CD123+), conventional DC type 1 (Lin-HLA-DR+CD11c+CD1c+CD141-), and conventional DC type 2 (Lin-HLA-DR+CD11c+CD1c-CD141+), (D) NK and NK CD56bright cells, (E) B cells and plasmabasts, (F) total TCR-γδ T cells, (G) MAIT (MR1+TCR-Vα7.2+) cells, (H) iNKT cells, (I) total α/β T cells, (J) α/β CD4+ T cells, (K) α/β CD8+ T cells and (L) α/β DNT cells (CD3+CD4−CD8−). Nonparametric Mann-Whitney tests were used for analysis, with $*P < 0.05$, $**P < 0.01$, $***P < 0.001$, and $****P < 0.0001$.

Supplemental Figure 5

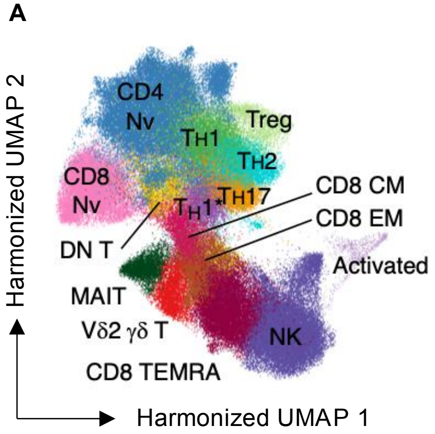

Supplemental Figure 5. Related to Figure 5

Single-cell transcriptomic analysis.

(A) Unsupervised clustering followed by manual identification with the aid of the SingleR pipeline guided by the MonacoImmuneDataset.

Supplemental Figure 6

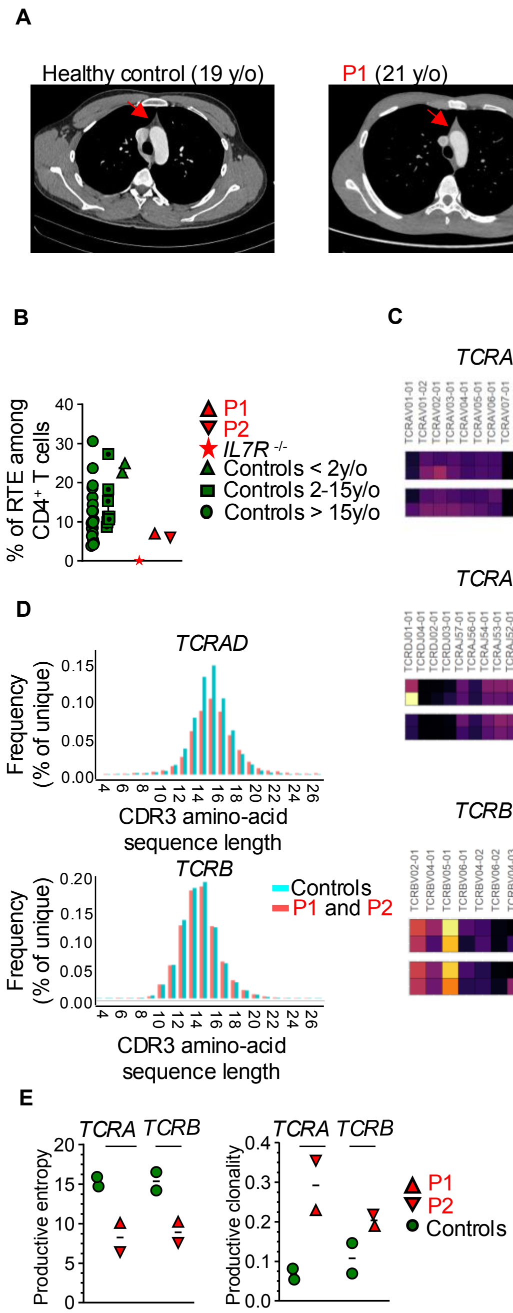

Supplemental Figure 6. Related to Figure 6

Impaired early T-cell development in AR IL-7 deficiency.

(A) CT scans for P1, P2, and P3. The thymic lodge, located between the sternum and the aortic arch, appears empty in P2 and P3 (56 years old), whereas a remnant thymus is visible in P1 (20 years old). (B) Frequency of recent thymic emigrant cells (RTE) defined as CD3+/CD4+/CD45RA+/CD31+ cells. (C) V and J gene usage for TCRA and V gene usage for TCRB in P1 and P2 relative to two age-matched controls. (D) CDR3 length distribution. comparison of CDR3 length distributions for the TCRA (upper) and TCRB (bottom) loci for two IL-7-deficient patients (P1 and P2, red) and two age-matched controls (blue). (E) Gini-Simpson index and Simpson clonality of TCRA and TCRB rearrangements in whole blood from P1, P2 and two age-matched healthy controls.

Supplemental Figure 7

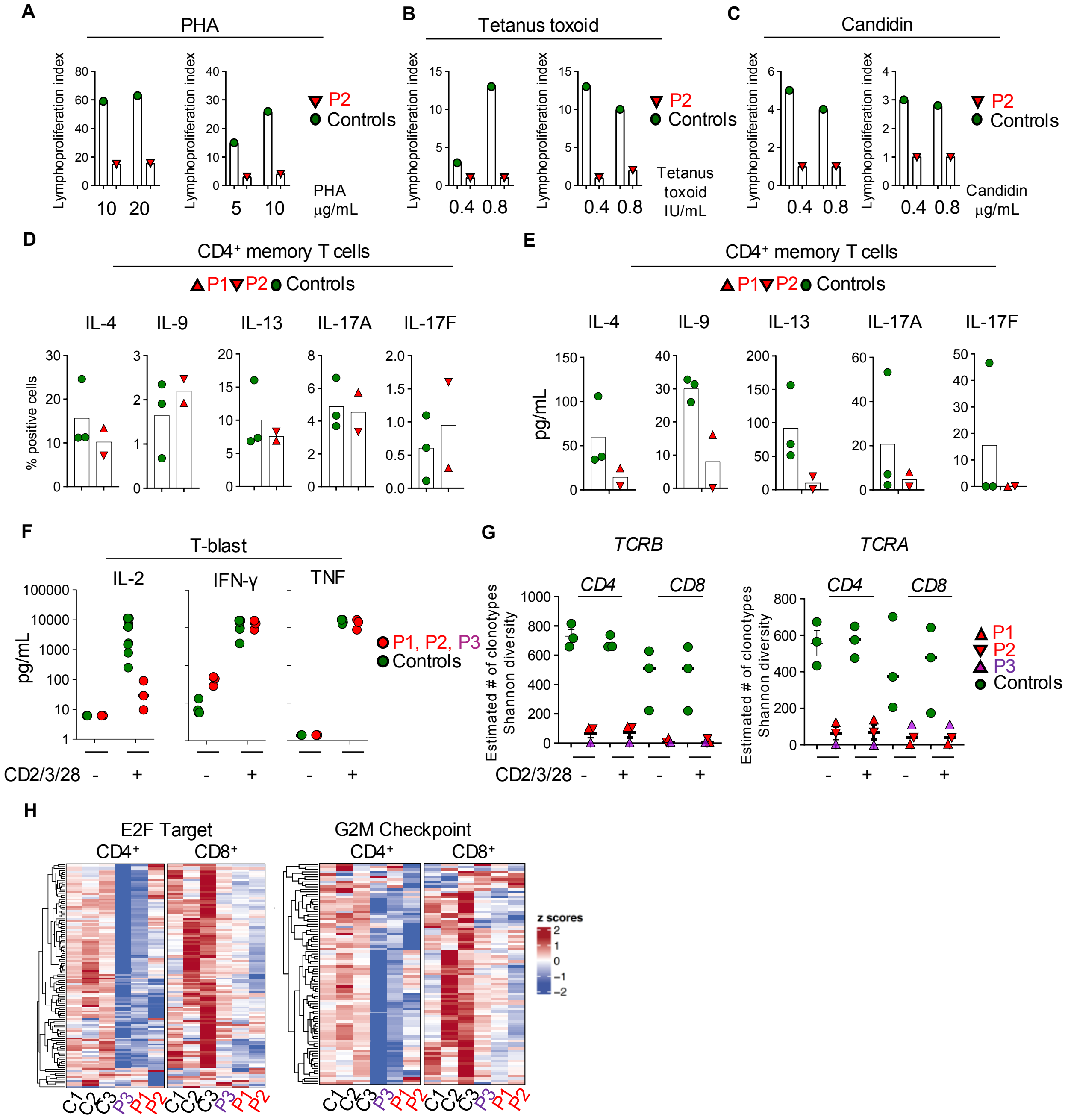

Supplemental Figure 7. Related to Figure 7

Peripheral T-cell functions.

(A-C) Fresh PBMCs from one age-matched control and P2 (obtained at the ages of 34 and 39 years, respectively), were incubated for 4 days with (A) PHA (5, 10 or 20 µg/mL), (B) tetanus toxoid (0.4 and 0.8 IU/mL) or (C) candidin (0.4 and 0.8 IU/mL). These experiments were performed twice (1998 and 2003). Histograms show the CFSE dilution for CD3+ T lymphocytes. (D-E) Memory CD4+ T cells from healthy controls (green dots), P1, and P2 were stimulated with PMA + ionomycin after five days of culture; the percentages of cells expressing IL-4, IL-9, IL-13, IL-17A, and IL-17F intracellularly were assessed by flow cytometry (D) and secreted cytokines were assessed by LEGENDplex (E). (F) Production of IL-2, IFN-γ, and TNF by T-cell blast lymphocytes after stimulation with anti-CD2/CD3/CD28 mAb cocktail for 6 hours. (G-H) Bulk RNA transcriptome analysis in sorted stimulated (2 h with anti-CD2/CD3/CD28 mAb cocktail) or non-stimulated CD4+ and CD8+ T-cell blasts from P1, P2, and P3, and three adult controls. (G) MiXCR analysis, (H) Geneset enrichment analysis (GSEA) for E2F and G2M checkpoint target genes. The heatmap shows adjusted z score values for each sample as a color gradient from blue for transcripts that were detected but below our significance cutoff values (downregulated), through purple, to red for adjusted z score values above our significance cutoff values (upregulated).

Supplemental Figure 8

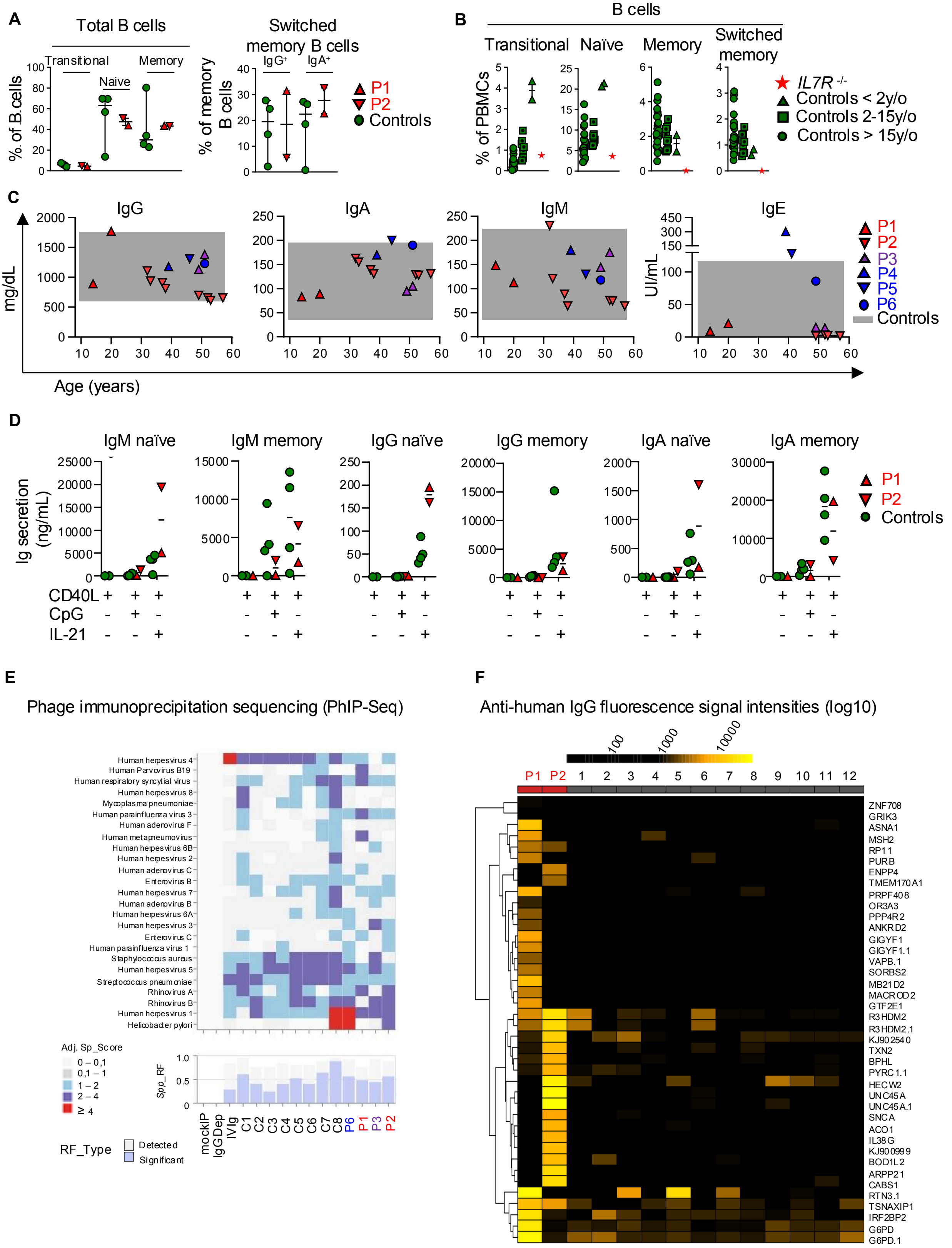

Supplemental Figure 8.

Preserved B-cell numbers and function in patients with AR IL-7 deficiency.

(A) Frequency of transitional, naïve, and memory B cells among total B cells, and switched memory B cells among memory B cells, in P1 (red triangle), P2 (inverted red triangle) and controls (green circles). (B) Frequency of transitional, naïve, memory, and switched memory B cells, from a four-month-old IL-7R-deficient SCID patient (red star), healthy controls under the age of two years (green triangles), healthy controls aged 2 to 15 years (green square), and healthy controls > 15 years old (green dots). (C) Follow-up of immunoglobulin levels (IgG, IgA, IgM, and IgE) in whole-blood samples from the patients (P1 red triangle, P2 inverted red triangle, P3 purple triangle, P4 blue triangle, P5 reverse blue triangle, P6 blue circle) and upper and lower limits of the normal range for age (gray). (D) IgM, IgG, and IgA secretion by naïve and memory B cell after stimulation with CD40L, alone or in combination with CpG, or IL-21 in P1 (red triangle), P2 (inverted red triangle) and controls (green circles). (E) Virscan. Adjusted virus scores for the indicated samples from P1, P2, P3, P6 and controls, mock IP samples and IgG-depleted serum, and IVIg. The heatmap shows adjusted virus score values for each sample as a color gradient from blue if antibodies were detected but below significance cutoff values, through purple, to red if the adjusted virus score values were above significance cutoff values. The bar plot (bottom) illustrates the size of the Ab repertoire for a given sample, indicating the precise number of different species for which peptide enrichment was observed (light blue) and the number of different species for which the adjusted virus score value exceeded the cutoff value for significance (dark blue). (F) Top hits from protein microarray-based autoantibody screening. Anti-human IgG fluorescence signal intensities (log10) for P1 and P2 and 12 controls plotted with correlation clusters for proteins, including the 40 proteins with the largest positive fold-change. Case-control status is marked at the top.

Supplemental Figure 9

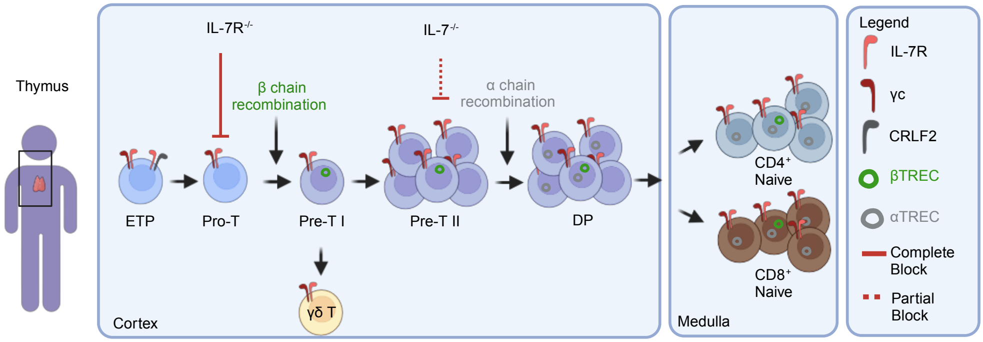

Supplemental Figure 9.

Schematic view of human T-cell development within the thymic cortex and medulla.

ETP is the most immature stage, and CD4+ or CD8+ T cells are the most mature T cells. Red lines identify developmental blocks in human AR IL-7R deficiency and red dashed lines identify developmental blocks in AR IL-7 deficiency. ETP: early thymic progenitor, DP: double-positive, SP: single-positive.

Supplemental Table 1: Overview of the clinical and genetic features of six patients with AR IL-7 deficiency

Kindred (Patient)	Sex	Origin	Year of birth (Age, years)	Last follow-up	*IL7* variants (cDNA protein)	Cutaneous warts HPV genotype (Age at onset)	Other viral diseases	Fungal diseases	Mycobacterial diseases	Vaccination	Others
**A** (P1)	M	Colombia	2001 (23)	Alive	c.284del/ c.284del p.N95Ifs*11/ p.N95Ifs*11	Common warts α-HPV2 (Since age 12)	-	Paranasal-sinus histoplasmosis (Age 3) Disseminated histoplasmosis (Age 20) Good response to systemic antifungal treatment	Pulmonary *Mycobacterium tuberculosis* (Age 8 and 12) Brain tuberculoma (age 12) Good response to anti-TB treatment Quantiferon TB: Negative	BCG (no AR)<br>COVID-19 (Once, no AR)	-
**B** (P2)	F	Colombia	1964 (60)	Alive	c.284del/ c.284del p.N95Ifs*11/ p.N95Ifs*11	Common and flat warts α-HPV2 (Since age 7)	Recurrent herpes zoster (Age 7, 28, 30-40) Recurrent	*Cryptococcus* meningitis (Age 28) Good response to systemic	Pulmonary mycobacterial disease *Mycobacterium spp.* (Age 30) Good response to anti-TB treatment Quantiferon TB: Negative	BCG (no AR)<br>COVID-19 (Twice, no AR)	-

							herpes simplex labialis (from age 30) COVID-19 no complications	antifungal treatment			
**C** (P3)	F	Japan	1966 (58)	Alive	c.3G>A/ c.3G>A p.M1?/ p.M1?	Common and flat warts α-HPV7 (Since age 9)	-	-	-	BCG (no AR)<br><br>COVID-19 (4 times, no AR)	Cutaneous SCC<br><br>Anti-phospholipid syndrome
**D** (P4)	M	Israeli/Arab	1964 (60)	Alive	c.205A>T / c.205A>T p.R69*/ p.R69*	Common and flat warts α-HPV3 (Since age 20)	Shingles (Age 55)	Cryptococcus meningitis (Age 20)	-	BCG (no AR)<br><br>COVID-19 (3 times, no AR)	Gastric diffuse large B-cell lymphoma (Age 54), CHOP and rituximab, in remission multiple SCC
**D** (P5)	F	Israeli/Arab	1969 (55)	Alive	c.205A>T / c.205A>T p.R69*/ p.R69*	Common and flat warts α-HPV-3 (Since age 20)	-	Cryptococcus meningitis (age 20)	-	BCG (No AR)	-
**D** (P6)	F	Israeli/Arab	1976 (48)	Alive	c.205A>T / c.205A>T	Common warts α-HPV-3 (Since age 20)	COVID-19 no	-	-	BCG (No AR)	-

					p.R69*/ p.R69*		complic ations				

Overview of the main demographic and clinical features of the six patients included in this study presenting recurrent cutaneous warts, fungal, and mycobacterial diseases. M: male, F: female, AR: adverse reaction, SCC: squamous cell carcinoma, BCC: basal cell cancer, ND: not determined, ages are indicated in years.

Supplemental Table 2: Cell counts in the peripheral blood of the six patients with AR IL-7 deficiency

**Patient**	**P1**	**P2**	**P3**	**P4**	**P5**	**P6**
**Age at investigation (years)**	19	56	54	56	51	44
**Sex**	M	F	F	M	F	F
**WBC (µL)** (4000-10000 c/mm^3)	5280	4455	4600	4100	2700	5160
**Total lymphocytes** (1200-4100 c/mm^3)	478	389	782	600	300	400
**$CD3^+$** (780-3000 c/mm^3)	228	138	483	378	107	166
**CD3+/CD4+** (500-2000 c/mm3)	47	18	28	48	31	36
**$CD3^+/CD8^+$** (200-1200 c/mm^3)	127	67	112	310	94	108
**γδ T cells** (25-200 c/mm^3)	52	60	ND	79	15	16
**Treg** (% of CD4 T cells) (3-8 %)	8	16	ND	13	6	4
**B cells** (64-820 c/mm^3)	73	38	266	83	66	48
**NK cells** (100-1200 c/mm^3)	121	171	ND	130	118	101

Monitoring of counts for leukocytes, T lymphocytes ($CD3^+$, $CD3^+/CD4^+$, $CD3^+/CD8^+$, γδ, Tregs), B lymphocytes, and NK cells in the blood of the patients. ND: not determined. In brackets are the reference values for lymphocyte subsets from Schatorjé EJH, et al. Scand. J Immunol. 2012 vol. 75 (4) pp. 436–44

Supplemental Table 3: Serological results for four patients with AR IL-7 deficiency

**Patient** (age at investigation, years)	**P1** (19)	**P2** (56)	**P3** (54)	**P4** (56)	**Threshold**
IgG anti-HSV-1	46	52	78.9	>1.1	>1.1
IgG anti-VZV	363	373	40.7	>135	>135
IgG anti-EBV	Present	Present	Present	ND	
IgG anti-CMV	169	88	1176	ND	>14
IgG anti-measles	81	>300	123	ND	>14
IgG anti-mumps	176	271	ND	ND	>11
IgG anti-rubella	190	54	>128	>10	>10
IgG anti-HAV	9	8	ND	>1	>1
IgG anti-tetanus toxoid (IU/mL)	0.32	0.15	ND	ND	>1 IU/mL = booster in five years
IgG anti-diphtheria toxoid (IU/mL)	0.15	<0.1	ND	ND	>1 IU/mL = booster in five years
IgG anti-*Haemophilus* (mg/L)	0.21	1.83	ND	ND	> 1 mg/L = protected
Serum anti-HIV antibodies (ELISA)	Negative	Negative	Negative	ND	
Anti-pneumococcal polysaccharide IgG	ELISA (mg/L)	ELISA (mg/L)	ND	ND	Protected if >1 mg/L
Serotype 4	0.38	0.15	ND	ND	Protected if >1 mg/L
Serotype 6B	0.52	1	ND	ND	Protected if >1 mg/L
Serotype 9V	0.27	0.4	ND	ND	Protected if >1 mg/L

Serotype 14	2.6	0.85	ND	ND	Protected if >1 mg/L
Serotype 18C	1.5	2.5	ND	ND	Protected if >1 mg/L
Serotype 19F	0.39	0.74	ND	ND	Protected if >1 mg/L
Serotype 23F	0.43	0.24	ND	ND	Protected if >1 mg/L

ND: not determined, HSV: herpes simplex virus, VZV: varicella zoster virus, EBV: Epstein-Barr virus, CMV: cytomegalovirus, HAV: hepatitis A virus, HIV: human immunodeficiency virus.

Supplemental Table 4: Cellular phenotypes of IL-7- and IL-7R-deficient humans relative to IL-7- and IL-7R-deficient mice and mice deficient for TSLP and CRLF2

	**Humans**		**Mouse model**			
	**IL-7 deficient**	**IL-7R deficient**	**IL-7 deficient**	**IL-7R deficient**	**TSLP deficient**	**CRLF2 deficient**
**Main phenotype**	CID $T^{low}B^{+}NK^{+}$	SCID $T^{-}B^{+}NK^{+}$	Low T- and B-cell counts, normal NK cell counts	Low T- and B-cell counts, normal NK cell counts	Normal T-, B-, and NK cell development	Normal T-, B-, and NK cell development
**αβ T cells**	Decreased	Decreased (Puel et al, 1998) (Giliani et al, 2005)	Decreased (von Freeden-Jeffry et al, 1995; Moore et al, 1996)	Decreased (Peschon et al, 1994)	Normal (Eckhardt J. et al, 2015 ; Reardon et al, 2011)	Normal (Carpino et al, 2004; Al-Shami, et al, 2004)
**CD4:CD8 ratio**	Inverted	Inverted (Puel et al, 1998)	Normal distribution (von Freeden-Jeffry et al, 1995)	Normal distribution (Peschon et al, 1994)	Normal (Eckhardt J. et al, 2015)	Normal (Al-Shami et al, 2004)
**Thymocytes**	Peripheral DNT: Normal<br><br>Peripheral DPT: Decreased	Peripheral DNT: Decreased<br><br>Peripheral DPT: Strongly decreased	Partial block from DN2 to DN3 Partial inhibition of the differentiation of $CD44^{+}$ $CD25^{+}$ ProT cells into $CD44^{-}$ $CD25^{+}$ pre-T cells	Strong block from DN1 to DN2 Partial inhibition at the earliest stage, $CD44^{+}$ $CD25^{-}$ pre-ProT cells into $CD44^{+}$ $CD25^{+}$ pro-T cells (Peschon et al, 1994)	DN1: Normal	Normal (Carpino et al, 2004; Al-Shami et al, 2004)

			(Moore et al, 1996) (Boudil et al, 2015) (Chappaz et al, 2007)<br><br>DN1: Decreased<br>DN2: Decreased<br>DN3: Decreased<br>DN4: Decreased<br>DP: Decreased<br>CD4 SP: Decreased<br>CD8 SP: Decreased<br>(Jensen et al, 2008)	DN1: Strongly Decreased<br>DN2: Decreased<br>DN3: Strongly Decreased<br>DN4: Strongly Decreased<br>DP: Strongly Decreased<br>CD4 SP: Strongly Decreased<br>CD8 SP: Strongly Decreased<br>(Jensen et al, 2008)	DN2: Normal<br>DN3: Normal<br>DN4: Normal<br>DP: Normal<br>CD4 SP: Normal<br>CD8 SP: Normal<br>(Jensen et al, 2008)	DN1: Normal<br>DN2: Normal<br>DN3: Normal<br>DN4: Normal<br>DP: Normal<br>CD4 SP: Normal<br>CD8 SP: Normal<br>(Jensen et al, 2008)
**ɣδ T cells**	Normal	Decreased *	Absent, block Immature $V\gamma3^{low}CD24^{+}$ to mature $V\gamma3^{high}CD24^{-}$ (Moore et al, 1996)	Absent (Maki K et al, 1996; He Y.W. & Malek T.R. 1996)		Normal (Al-Shami et al, 2004)
**B cells**	Slightly low or normal numbers	Normal numbers (Puel et al, 1998) (Giliani et al, 2005)	Decreased and abnormal B-cell population $B220^{+}$, IgM^{-} (von Freeden-Jeffry et al, 1995)	Strongly decreased (Peschon et al, 1994) (Voβhenrich et al, 2003)		Normal (Carpino et al, 2004; Al-Shami, et al, 2004)

**B-cell development or peripheral cell proportions**	**Transitional:** Normal proportions **Naïve:** Normal proportions **Memory:** Normal proportions **Switched memory B cells:** Normal proportions	Impaired differentiation during the early phases of B lymphopoiesis (High levels of CLPs, pro-B cells; Fewer pre-BI cells) suggesting arrest at the pro-B to pre-BI cell transition. (Kaiser et al, 2023)<br><br>**Transitional:** Normal proportions **Naïve:** Normal proportions **Memory:** Absent **Switched memory B cells:** Absent (Kaiser et al, 2023)	Blocked Pro-B to Pre-B (Fraction B/C to Fraction D) (von Freeden-Jeffry et al, 1995)	Blocked Pre-Pro B to Pro-B (Fraction A to Fraction B) (Peschon et al, 1994)		Normal (Carpino et al, 2004; Al-Shami et al, 2004)
**NK cells**	Normal numbers	Normal (Puel et al, 1998)	Normal numbers to a modest decrease (Moore et al, 1996)	Normal (He Y.W. & Malek T.R.1996)		

**Secondary lymph organs**	No palpable lymph nodes (P1–P4)<br><br>Remnant thymus (P1)<br><br>Presence of tonsil (P2)	Thymus: Hypocellular and not well structured<br><br>Lymph nodes: Hypocellular and not structured, no evidence of germinal centers<br><br>Necropsy (Zago et al, 2014)	Lymph nodes and Peyer's patches: Absent (von Freeden-Jeffry et al,1995)<br><br>Thymus and spleen: small size, low weight and cellularity (von Freeden-Jeffry et al, 1995) (Chappaz et al, 2007) Diminished entry of B and T cells into LN in adult mice with IL7 depletion (Yang et al, 2018)	Lymph nodes: Hypocellular or absent (Seymour et al, 2006; Luther et al, 2003)<br><br>Peyer's patches: absent (Adachi S, et al 1998)<br><br>Thymus and spleen: low cellularity (Peschon et al, 1994; Erlandsson et al, 2004)	Normal (Reardon et al, 2011)	Normal (Carpino et al, 2004; Al-Shami et al, 2004)

NR: not reported, CID: combined immunodeficiency, SCID: severe combined immunodeficiency, DNT: double-negative T cells, NK: natural killer cells,
IL-7: interleukin 7, IL-7R: interleukin 7 receptor, TSLP: thymic stromal lymphopoietin, CRLF2: cytokine receptor like factor 2, CLPs: common lymphoid progenitors, LN: lymph nodes.

Supplemental Table 5: Oligonucleotides used for TREC quantification

**TRECs**	
Sj-In3	GTGCTGGCATCAGAGTGTGT
Sj-In5	TGATGCCACATCCCTTTCAA
Sj-Out3	ACACTTGCTCCGTGGTCTGT
Sj-Out5	CTCTCCTATCTCTGCTCTGAA
CD3-out3	AGCTCTGAAGTAGGGAACATAT
CD3-out5	ACTGACATGGAACAGGGGAA
CD3-in3	TTCCTGGCCTATGCCCTTTT
CD3-in5	GGCTATCATTCTTCTTCAAGGTA
