# Supplementary material for: IL-7–dependent and –independent lineages of IL-7R–dependent human T cells
Source: J Clin Invest. 2024 Oct 1;134(19):e180251. doi: 10.1172/JCI180251 (PMC11444196; doi:10.1172/JCI180251)

Figure 2

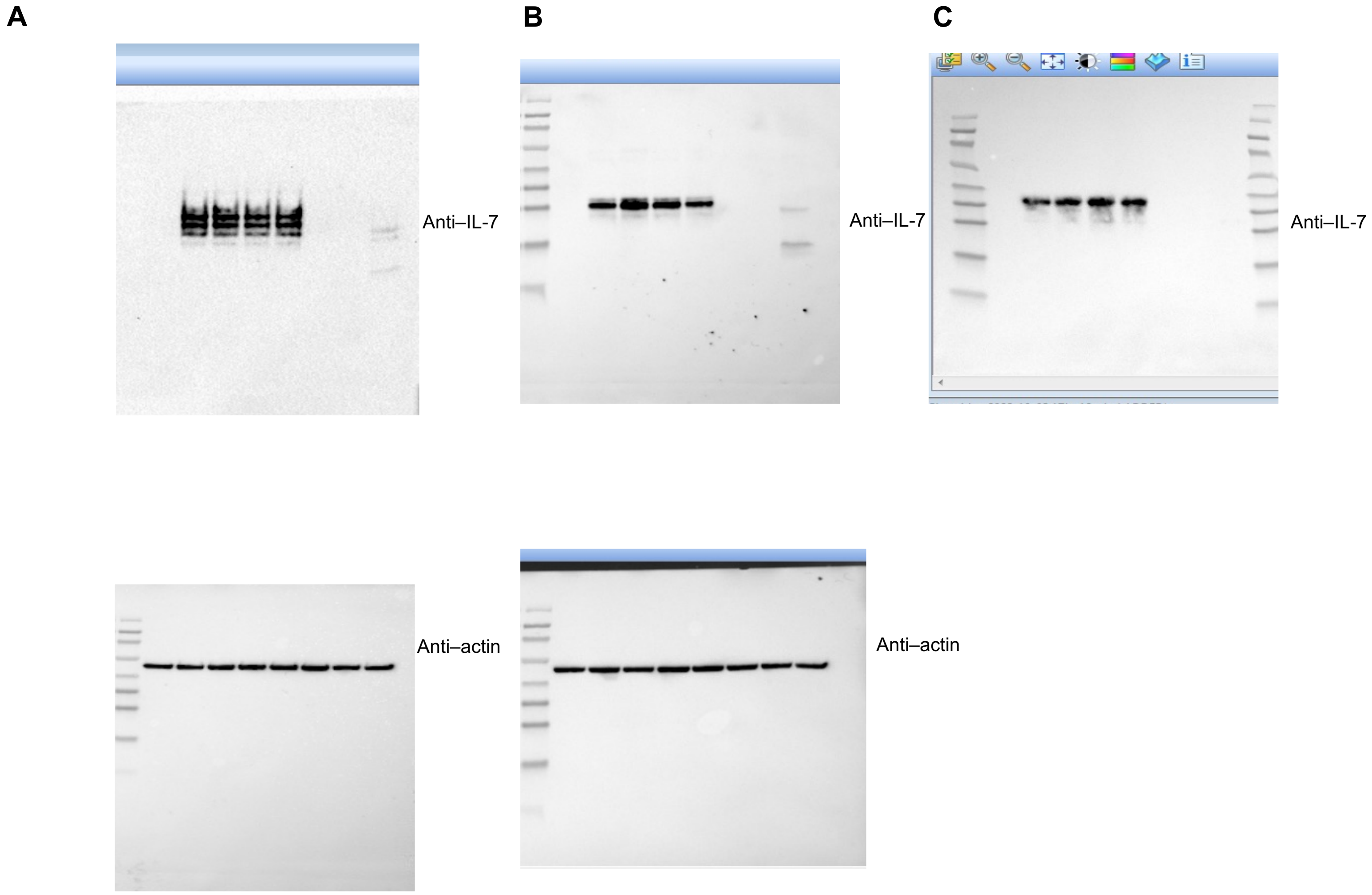

Supplemental Figure 2

B

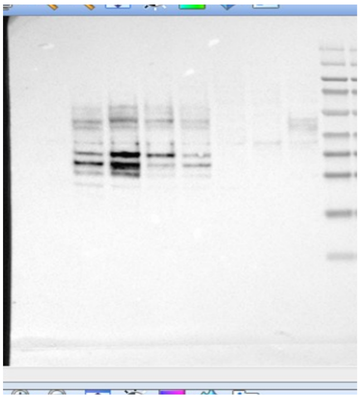

Anti-DDK

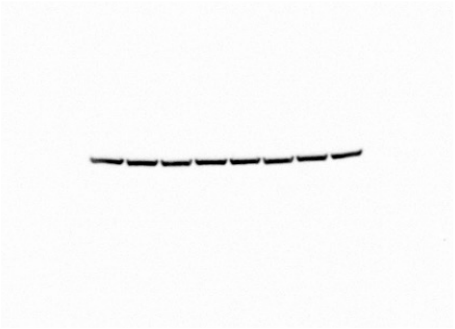

Anti-actin

C

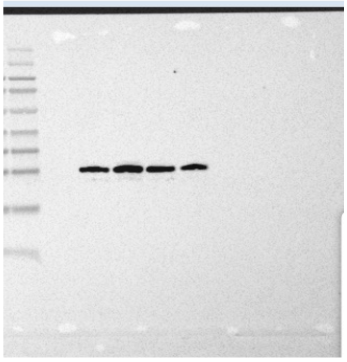

Anti-DDK

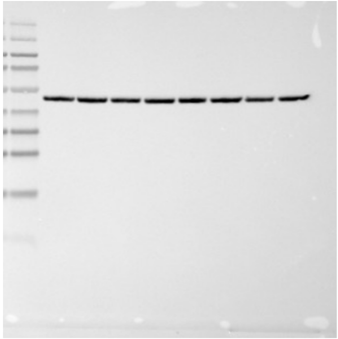

Anti-actin

Supplemental Figure 3

D E

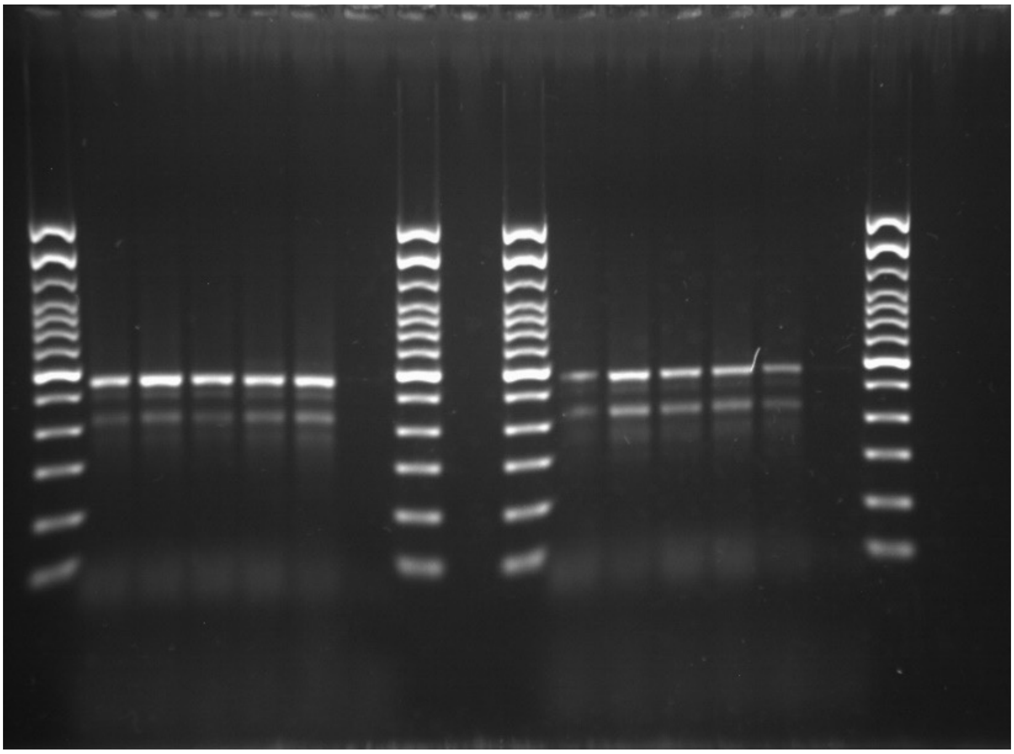

Supplement: Unedited blot and gel images [file jci-134-180251-s158.pdf]
